# A novel miR-365-3p/EHF/keratin 16 axis promotes oral squamous cell carcinoma metastasis, cancer stemness and drug resistance via enhancing β5-integrin/c-met signaling pathway

**DOI:** 10.1186/s13046-019-1091-5

**Published:** 2019-02-19

**Authors:** Wei-Chieh Huang, Te-Hsuan Jang, Shiao-Lin Tung, Tzu-Chen Yen, Shih-Hsuan Chan, Lu-Hai Wang

**Affiliations:** 1 Institute of Molecular Medicine, National Tsing Hua University, Hsinchu, 300 Taiwan; 2 Chinese Medicine Research Center, China Medical University, Taichung, 404 Taiwan; 3 Research Center for Chinese Herbal Medicine, China Medical University, Taichung, 404 Taiwan; 4 Department of Hematology and Oncology, Ton-Yen General Hospital, Hsinchu, 302 Taiwan; 5 Center for Advanced Molecular Imaging and Translation (CAMIT), Chang Gung Memorial Hospital, Taoyuan, 333 Taiwan; 6 Department of Nuclear Medicine, Chang Gung Memorial Hospital, Taoyuan, 333 Taiwan; 7 Graduate Institute of Integrated Medicine, China Medical University, Taichung, 404 Taiwan

**Keywords:** Oral squamous cell carcinoma, miR-365-3p, Keratin 16, C-met, ETS homologous factor, β5-integrin, Chemoresistance

## Abstract

**Background:**

Targeting the c-Met signaling pathway has become a therapeutic strategy in multiple types of cancer. We unveiled a novel c-Met regulating mechanism that could be applied as a modality for oral squamous cell carcinoma (OSCC) therapy.

**Methods:**

Upregulation of keratin 16 (KRT16) was found by comparing isogenic pairs of low and high invasive human OSCC lines via microarray analysis. OSCC cells with ectopic expression or silencing of KRT16 were used to scrutinize functional roles and associated molecular mechanisms.

**Results:**

We observed that high KRT16 expression significantly correlated with poorer pathological differentiation, advanced stages, increased lymph nodes metastasis, and decreased survival rate from several Taiwanese OSCC patient cohorts. We further revealed that miR-365-3p could target ETS homologous factor (EHF), a KRT16 transcription factor, to decrease migration, invasion, metastasis and chemoresistance in OSCC cells via inhibition of KRT16. Under confocal microscopic examination, c-Met was found possibly partially associates with KRT16 through β5-integrin. Colocalization of these three proteins may facilitate c-Met and β5-integrin–mediated signaling in OSCC cells. Depletion of KRT16 led to increased protein degradation of β5-integrin and c-Met through a lysosomal pathway leading to inhibition of their downstream Src/STAT3/FAK/ERK signaling in OSCC cells. Knockdown of KRT16 enhanced chemosensitivity of OSCC towards 5-fluorouracil (5-FU). Various combination of c-Met inhibitor (foretinib), protein tyrosine kinase inhibitor (genistein), β5-integrin antibody, and 5-FU markedly augmented cytotoxic effects in OSCC cells as well as tumor killing effects in vitro *and* in vivo*.*

**Conclusions:**

Our data indicate that targeting a novel miR-365-3p/EHF/KRT16/β5-integrin/c-Met signaling pathway could improve treatment efficacy in OSCC.

**Electronic supplementary material:**

The online version of this article (10.1186/s13046-019-1091-5) contains supplementary material, which is available to authorized users.

## Background

Oral squamous cell carcinoma (OSCC) is the sixth most common cancer worldwide and accounts for more than 95% of head and neck cancers [1, 2]. The incidence of OSCC in Taiwan has increased by 30% in the last 5 years, and the mortality rate has increased by 25% [2]. The behavior of OSCC is aggressive with the propensity for local recurrence and distant metastasis due to innate and acquired chemoresistance resulting in adverse prognosis [3, 4]. To overcome chemoresistance to improve treatment efficacy in OSCC is urgent and indispensable.

Keratins are intermediate filament family proteins expressed in epithelial cells and are obligate heteropolymers between a type I and a type II protein. Keratin 6 (KRT6) and keratin 16 (KRT16) have been identified in the epidermis and cells of all stratified squamous epithelia, but are also expressed in ductal luminal cells and in secretory cells of human eccrine sweat glands [5]. Some studies have implied the role of keratins in tumorigenesis and metastasis [5, 6], and overexpression of keratin 8, 18, and 19 is often observed in poorly differentiated OSCC [7, 8]. In addition, keratins associate with integrins and interact with hemidesmosomes (HDs), which are vital for cell-matrix adhesion and migration [9, 10], and a study reported that keratins were associated with α6β4-integrin through plectin and dystonin and that they played a role in promoting cancer cell properties [11]. Keratins stabilize HDs by regulating integrins and extracellular matrix molecules, suggesting that they might also control cancer progression by enhancing integrin signaling function in tumor cells [10, 12]. Although keratins play a role in cancer progression, their precise act and signaling pathways in regulating cancer stemness and metastasis, particularly in OSCC, remain unclear.

Integrins are a family of transmembrane glycoproteins that form heterodimeric receptors for extracellular matrix molecules and mediate cell–matrix and cell–cell interactions [13]. Signal transduction generated by integrins was reported to regulate gene expression, tumor progression, cancer stemness, and metastasis [14, 15]. Moreover, increased expression of certain integrins within the primary tumor is associated with enhanced metastasis and an adverse prognosis in numerous cancers [15]. Previous report identified that keratins associated with β4-integrin through plectin in an HD complex [16]. However, the functional role of keratins-integrins complexes and their link with c-Met in OSCC is still unknown.

In this study, we found upregulation of KRT16 in highly invasive OSCC lines and in OSCC tumor tissues. We also observed that high KRT16 expression was significantly correlated with advanced stages and poor clinical outcomes. We further identified that miR-365-3p targeted EHF, leading to KRT16 depletion as well as increased lysosomal degradation of β5-integrin and c-Met in OSCC cells. Furthermore, we unveiled the novel miR-365-3p/EHF/KRT16/β5-integrin/c-Met pathway capable of regulating OSCC cell migration, invasion, metastasis, and cancer stemness by activation of the Src/STAT3 signaling. Treatment with inhibitor(s) targeting this pathway including KRT16-siRNA, c-Met inhibitor (foretinib), protein tyrosine kinase inhibitor (genistein), and β5-integrin antibody (ITGB5-ab) in combination with a commonly used chemotherapeutic agent, 5-fluorouracil (5-FU), in OSCC resulted in significantly enhanced cytotoxicity and tumor killing effects in OSCC cells. Thus, targeting this novel pathway provides a new therapeutic approach for OSCC.

## Methods

### Acquisition of tissue samples

All clinical samples were acquired through protocols approved by the respective institutional review boards. All clinical tumor tissues including 24 normal oral and tumor tissue pairs, 56 oral cancer samples for cDNA microarray analysis, and 294 OSCC tissue microarray slides were obtained from the Chang Gung Memorial Hospital-Linkou in Taiwan. Normal and tumor oral tissue samples from surgical resection were snap-frozen in liquid nitrogen or embedded in RNA later. A commercial tissue array slide containing 144 normal and OSCC tissues was purchased from US Biomax Inc.

### Cell culture

Human OSCC cell lines including OC-3, CGHNC9, and C9-IV3 sublines have been described previously [17]. OC-3 and CGHNC9 lines were selected 1 to 3 cycles by in vivo injection of the respective cells into tail vein of C.B-17 severe-combined immunodeficiency (CB17-SCID mice), followed by isolation of tumor cells grown from lung metastases to obtain OC-3-IV, OC-3-IV2 (OC-3-IV-M) and CGHNC9-IV3 (C9-IV3) where the number following IV denotes the cycle of selection. CGHNC9 and C9-IV3 cells were grown in Dulbecco’s modified Eagle medium (DMEM; Invitrogen, Carlsbad, CA, USA) with 10% fetal bovine serum (FBS; Invitrogen, Carlsbad, CA, USA). The cells of OC-3 and its derived invasive sublines, OC-3-IV and OC-3-IV-M, were grown in a 1:1 mixture of DMEM and keratinocyte serum-free medium (KSFM; Invitrogen, Carlsbad, CA, USA) with 10% FBS. All cells were incubated at 37 °C in 5% CO_2_.

### Vectors, antibodies, and reagents

The luciferase-3′-untranslated region (3’UTR)-wild (wt) reporter or luciferase- 3’UTR-mutant (mt) plasmids were prepared by inserting the EHF-3’UTR-wt carrying a putative miR-365-3p binding site or its mutant sequence into the pGL3-control plasmid (sequences are shown in the Additional file 1: Table S1). OC-3-IV and OC-3-IV-M were transfected with pSuper-puro, pSuper-puro-shKRT16, or pSuper-puro-shEHF and clone selection was employed to screen for puromycin (Sigma-Aldrich) stably expressing cancer cell lines (Additional file 1: Figure S1a and S1b). OC-3-IV and OC-3-IV-M cell lines stably transfected with pPG-GFP-miR vector or pPG-GFP-miR-365-3p were established by selection with blasticidin (Sigma-Aldrich) (Additional file 1: Figure S1c). Four or five individual clones were mixed in equal proportion as stable clones. The detailed primers used for miRNAs expression are listed in the Additional file 1: Table S2. KRT16 and EHF coding sequences were cloned in the pcDNA3.1 plasmid. Antibodies for immunoblotting and immunohistochemistry (IHC) include anti-KRT16 and ErbB2 (Abcam), anti-ITGA4, anti-ITGA5, anti-ITGB1, anti-ITGB3, anti-ITGB4, anti-ITGB5, anti-Src, anti-p-Src-416, anti-p-Src-527, anti-STAT3, anti-p-STAT3–708, anti-AKT, anti-p-AKT-473, anti-Erk1/2, anti-p-Erk1/2, anti-EGF receptor (EGFR), anti-LC3, anti-JAK2, anti-p-c-Met (Cell Signaling Technology), anti-c-Met, anti-IGF-1R, anti-EHF, anti-actin (Santa Cruz Biotechnology, Inc.), anti p-FAK-397 (Gene Tex Inc.), and anti-FAK (Merck Millipore). EHF, KRT16, and β5-integrin siRNAs were purchased from MDBio, Inc. The sequences of EHF-, KRT16-, β5-integrin-, and c-Met-siRNA oligonucleotides are shown in the Additional file 1: Table S1. Treatments with KRT-16-siRNA, 10 μMMG132 (Sigma-Aldrich Inc., St Louis, MO, USA), and 100 nM bafilomycin A (B.A.; Sigma-Aldrich Inc., St Louis, MO, USA) were applied for 48, 8, and 24 h, respectively. OC-3-IV was transfected with NC-siRNA or KRT16-siRNA, and 20 ng/mL HGF (Sigma-Aldrich Inc., St Louis, MO, USA) was added to the cells for 30 min. For cell sensitivity assays, OC-3-IV and C9-IV3 cells were pretreated with 5-FU (Sigma-Aldrich Inc., St Louis, MO, USA), foretinib (Sigma-Aldrich Inc., St Louis, MO, USA), genistein (Sigma-Aldrich Inc., St Louis, MO, USA), and ITGB5-ab in serum-free culture medium for 18 h (overnight; O/N). Cells were transfected using the Lipofectamine 2000 (Invitrogen) and Lipofectamine RNAiMAX (Invitrogen).

### 3’ UTR luciferase reporter and luciferase promoter assay

In the 3’ UTR reporter assay, pGL3-EHF-3’UTR-wt or pGL3-EHF-3’UTR-mt was cotransfected with pre-miR-365-3p or pre-anti-miR-365-3p into CGHNC9 cells. In the promoter assay,OC-3-IV cells were transfected with pGL3-contorl-KRT16-2 k (− 2249/+ 5), − 1 k (− 938/+ 5), 0.5 k (− 522/+ 5), or 0.25 k (− 254/+ 5) promoter elements together with *Renilla*. Luciferase assay was conducted using an assay kit (Promega corporation, Madison, WI). *Renilla* luciferase was cotransfected as a control for normalization (Promega corporation, Madison, WI).

### Sphere-forming assay

Monolayer cells of OSCC cells were cultured in a stem cell selective condition described previously to obtain spheres [18]. Spheres comprised at least five cells were calculated according to a published report [19].

### RNA extraction and RT-PCR

Reverse transcriptase (RT)-polymerase chain reaction (PCR) and quantitative RT (qRT)-PCR were used to detect the miR-365-3p and mRNA expression. We designed a stem-loop RT primer to specifically hybridizing with miR-365-3p or RNU6B. RNU6B was used for normalization. This assay included a reverse transcription reaction using ReverTra Ace (TOYOBO, Osaka, JAPAN). RT-PCR and qRT-PCR were performed with a 1:10 dilution of cDNA, using KAPA SYBR FAST qPCR Kits (KAPA Biosystems, Wilmington, United States) and a qRT-PCR detection system (Bio-Rad, College Station, Texas, USA). The gene expression level was normalized using actin mRNA. The primers used for mRNA expression are listed in the Additional file 1: Table S2.

### Cell chemotactic migration and invasion assay

Detailed procedures were described elsewhere [17].

### In vivo metastasis assays and tumorigenicity experiments

OSCC cells were intravenously injected into the tail veins of 6–8-week-old CB17-SCID mice, which were then monitored for 30–60 days before sacrifice. Lung tissues were removed, fixed, paraffin-embedded, serially sectioned, and subjected to hematoxylin and eosin (H&E) staining. OC-3-IV parental cells and sphere cells were prepared, and a total of 1 × 10^3^ cells from each were injected individually into the buccal mucosa of 6–8-week-old CB17-SCID mice. Tumor growth was monitored through live animal bioluminescence imaging (BLI; Caliper IVIS system, Perkin Elmer). Mice were sacrificed 40–60 days after implantation. In the subcutaneous implantation model, OC-3-IV cells (1 × 10^6^) were harvested and resuspended in 20 mL of phosphate-buffered saline (PBS) containing 50% Matrigel (BD Biosciences). Cells were injected subcutaneously into the hind backs of 6–8-week-old CB17-SCID mice. In the experiments of treatment with 5-FU, foretinib, and ITGB5-ab (50 μg/mouse), 65 mg/mL 5-FU was dissolved in dimethyl sulfoxide (DMSO) and further diluted in ethylene glycol for an injection does of 12.5 mg/kg; 5 mg/mL of foretinib was dissolved in dimethyl sulfoxide (DMSO) and further diluted in ethylene glycol for an injection dose of 1.3 mg/kg. These preparations were subsequently orally or intravenously injected into mice twice weekly starting from day 14 after cell implantation.

### Immunohistochemistry (IHC) and immunofluorescence (IF) microscopy

IHC was conducted to detect KRT16 expression from paraffin-embedded oral cancer specimens. The slides were stained with primary antibody using an automatic slide stainer BenchMark XT (Ventana Medical Systems). The IHC score of KRT16 for each specimen was graded as follows: **+**, weak; **++**, moderate; **+++**, strong. Subsequently, 2.5 × 10^4^ OC-3-IV cells were seeded on polylysine-coated cover slips for 150 min in complete medium and then fixed with 4% formaldehyde for 5 min at room temperature prior to IF assay. The focal adhesion areas were measured by calculating the fluorescence areas using ImageJ software. Cells were transfected with the plasmids indicated in the figure legends for 6 h, and subsequently fixed with 4% formaldehyde for 5 min, washed three times with PBS, treated with 0.1% Triton for 10 min, and blocked with 5% goat serum for 1 h. The cells were subsequently incubated with vinculin antibody at 200× dilution at 4 °C O/N followed by binding with Alexa Flour 488 goat anti-rabbit for green fluorescence.

### Chromatin immunoprecipitation assay (ChIP)

ChIP material was prepared in accordance with the Magna ChIP (Millipore) manufacturer’s guidelines. Briefly, 1 × 10^7^ OC-3-IV cells were transfected with 10 μgpCDNA3.1 plasmid or EHF plasmid O/N, after which ChIP assay was performed. Immunoprecipitation (IP) was applied using 1 μg of EHF antibody (Santa Cruz Biotechnology) and an equivalent amount of rabbit IgG and H3 antibodies (Millipore). PCR primers were designed on the site-3 region of KRT16, and the primer sequences used are provided in the Additional file 1: Table S1. The PCR products were visualized through agarose gel electrophoresis or quantitative PCR. Experiments were repeated twice.

### Immunoblotting and co-immunoprecipitation (co-IP)

OSCC cells were lysed in radioimmune precipitation assay buffer (150 mM NaCl, 1% Nonidet P-40, 0.5% deoxycholic acid, 0.1% SDS, 50 mM Tris-HCl; pH 8.0) containing protease and phosphatase inhibitor mixture (Roche).

### Cell viability assay

The cell proliferation assay was evaluated using an MTT assay (Promega, Madison, WI, USA) according to the manufacturer’s protocol. Briefly, cells were plated at a density of approximately 3000 cells per well in 96-well plates and were incubated for 24 h. Cells were subsequently treated with different concentrations of 5-FU, foretinib, genistein, and ITGB5-ab, and then were incubated for 48 h. The quantity of formazan product, which is directly proportional to the number of viable cells, was measured at a wave length of 490 nm with a 96-well plate reader. The drug concentration required to suppress proliferation by 50% is defined as IC50. All data were calculated from three independent experiments performed in triplicate.

### Statistics

The GraphPad Prism software (GraphPad Software, CA) was used to generate graphs and two-tailed paired or unpaired *t*-tests were performed to determine the significance between the groups compared. Except where otherwise noted, data presented in figures are shown as mean ± standard deviation (SD). Survival data were analyzed using the Kaplan–Meier method. Differences between experimental groups were calculated using the log-rank test. Differences with *P* < 0.05 (*P < 0.05 and **P < 0.01) are considered statistically significant. The differences in the expression levels of KRT16 in the OSCC tissues of different stages were calculated using Fisher’s exact probability test. The differences in the expression levels of KRT16, β5-integrin, and c-Met in the normal and primary OSCC tissues were calculated using a paired *t*-test.

## Results

### Overexpression of KRT16 was found in highly invasive human OSCC cell lines and OSCC specimens with clinical significances

**Fig. 1:**
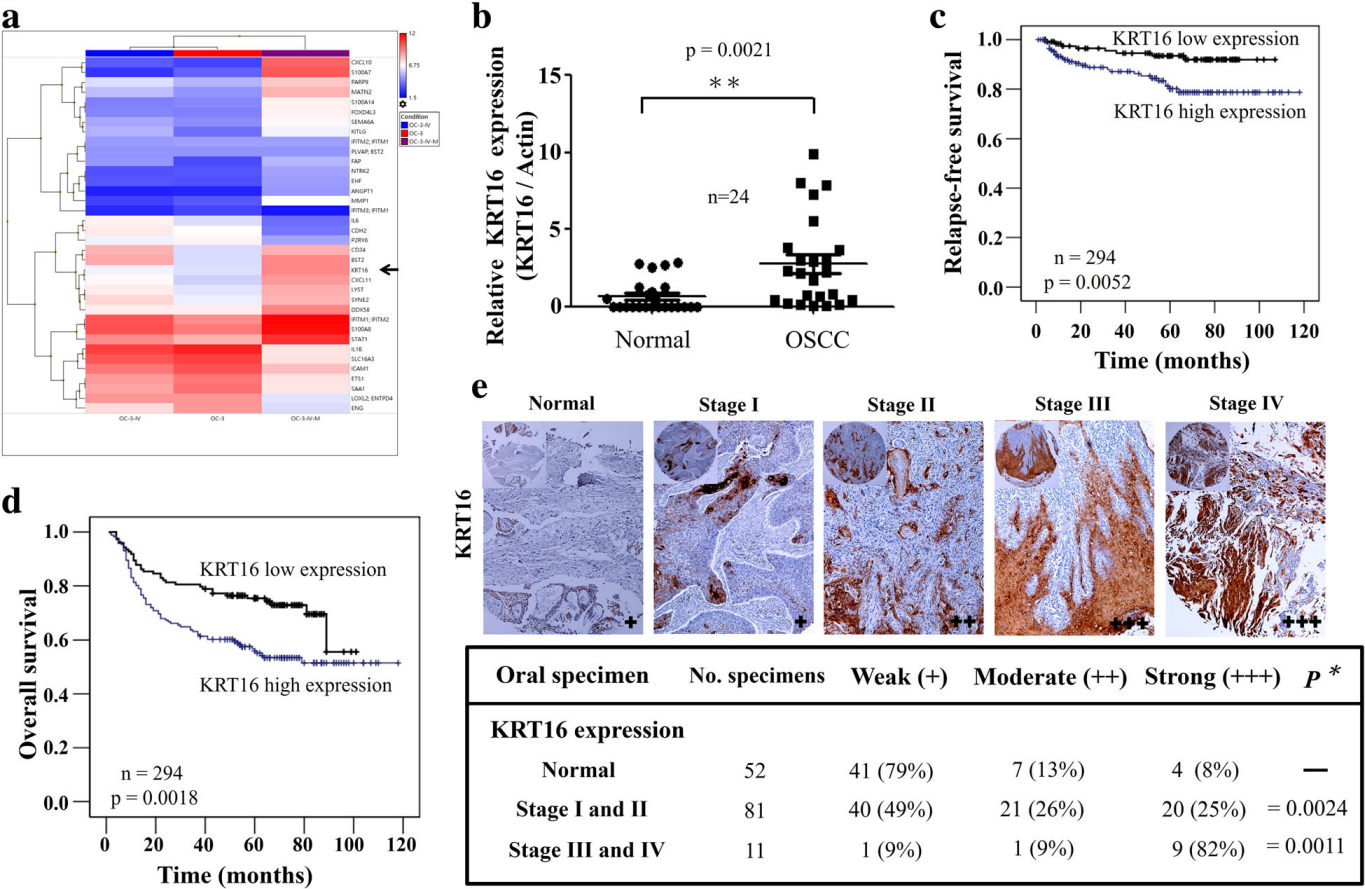
KRT16 expression and its clinical significances in OSCC. **a** Heat map of the 36 most differentially regulated genes from cDNA microarrays of OC-3-IV (left), OC-3 (middle) and OC-3-IV-M cells (right). **b** QRT-PCR of mRNAs revealed upregulation of KRT16 mRNA in OSCC tissues compared with their matched normal oral tissues (3.21 ± 0.23-fold change; *P* = 0.0021). The relative amount of KRT16 normalized to small nuclear RNU6B. RNA was calculated using the eq. 2^–ΔCt^, where Δ*C*_T_ = (*C*_TKRT16 RNA_ – *C*_TRNU6BRNA_). **c** Retrospective analysis of Kaplan-Meier plots for KRT16 expression in association with relapse-free survival of 294 patients. The level of KRT16 protein was determined through IHC. Tissues expressing KRT16 at levels lower than the median were assigned to the low expression group and those above the median level were assigned to the high expression group. **d** Retrospective analysis of Kaplan–Meier plots for KRT16 expression in association with overall survival of 294 patients. **e** Top, typical examples of the expression levels of KRT16 protein as determined through IHC staining in a commercial tissue array slide containing 144 normal and OSCC tissues. Bottom, the table shows the correlation between the KRT16 expression level and OSCC stages. For each section, staining was determined as weak (**+**), moderate (**++**), or strong (**+++**). Specimens were grouped as normal, stage I/II, and stage III/IV. *P* = 0.0024 for normal versus stage I/II, *P* = 0.0011 for stage I/II versus stage III/IV

To derive a highly invasive OSCC sublines, the established human OSCC line OC-3 cells were injected into tail veins of CB17-SCID mice. After one and two rounds of in vivo selection, we successfully isolated two highly invasive sublines, OC-3-IV and OC-3-IV-M respectively (Additional file 1: Figure S2). The OC-3-IV-M cells were found to have significantly increased migration, invasion and lung metastatic ability compared with the parental OC-3 or OC-3-IVcells (Additional file 1: Figure S2a, b and c). By analyzing the results derived from gene microarray analysis using Partek software and gene ontology program to compare the invasive phenotypes of the OC-3 vs. OC-3-IV, OC-3 vs. OC-3-IV-M and OC-3-IV vs. OC-3-IV-M (Fig. 1a, Additional file 1: Figure S3a, b and c), we found a series of genes with 4-fold or more differential expression between the OC-3 vs. OC-3-IV-M and OC-3-IV vs. OC-3-IV-M sublines. Six genes with the highest increase in expression were selected for validation through qRT-PCR (Additional file 1: Figure S4a). The three highest expressed genes including CXCL10, MMP1, and KRT16 were chosen for further analysis.

First, we started to examine 24 pairs of normal oral and cancer tissues to determine the expression of these three genes (Fig. 1b, Additional file 1: Figure S4b). Among these three genes, only KRT16 was significantly overexpressed in the 24 OSCC samples compared to their matched normal tissues (Fig. 1b). KRT16 was also found to possess an ascending trend of upregulation in OC-3-IV and OC-3-IV-M sublines (Additional file 1: Figure S3a, b and c). The expression of KRT16 increased by 1.31 fold and 8.65 fold in OC-3-IV and OC-3-IV-M sublines respectively when compared with parental OC-3 cells (Fig. 1a). Secondly, we analyzed the cDNA microarray data of 56 OSCC samples from a Taiwanese cohort series. We calculated the median KRT16 expression level of these samples, and thus divided them into high (above median) and low (below median) expression groups for Kaplan–Meier survival analysis. This analysis revealed that patients with high KRT16 expression were correlated with significantly poorer overall survival and more lymph node metastasis (Additional file 1: Figure S4c). Thirdly, we examined 294 clinical OSCC samples for KRT16 expression through IHC and found that patients with higher KRT16 expression had significantly poorer relapse-free and overall survival (Fig. 1c and d). Analysis of clinical parameters of those patients also revealed that KRT16 expression significantly correlated with moderate to poor pathological differentiation, advanced stages (stage III/IV), exposure to alcohol, betel nut, and cigarette, and disease relapse (Table 1). Finally, we examined KRT16 expression in a commercial tissue array slide containing 144 normal and OSCC specimens by IHC staining. OSCC specimens expressed higher KRT16 expressions than the normal tissues in all stages (Fig. 1e). The KRT16 expression level was higher in stage III/IV than in stage I/II of OSCC (Fig. 1e). In stage III/IV samples, approximately 91% cases had moderate to strong expression of KRT16, whereas only approximately 51% cases had moderate to strong expression in stage I/II samples (Fig. 1e). These findings indicated that overexpression of KRT16 was associated with poorer tumor differentiation, advanced stages, more lymph nodes metastasis, exposure to alcohol/betel nut/cigarette, and poor clinical survival in OSCC.

**Table 1:**
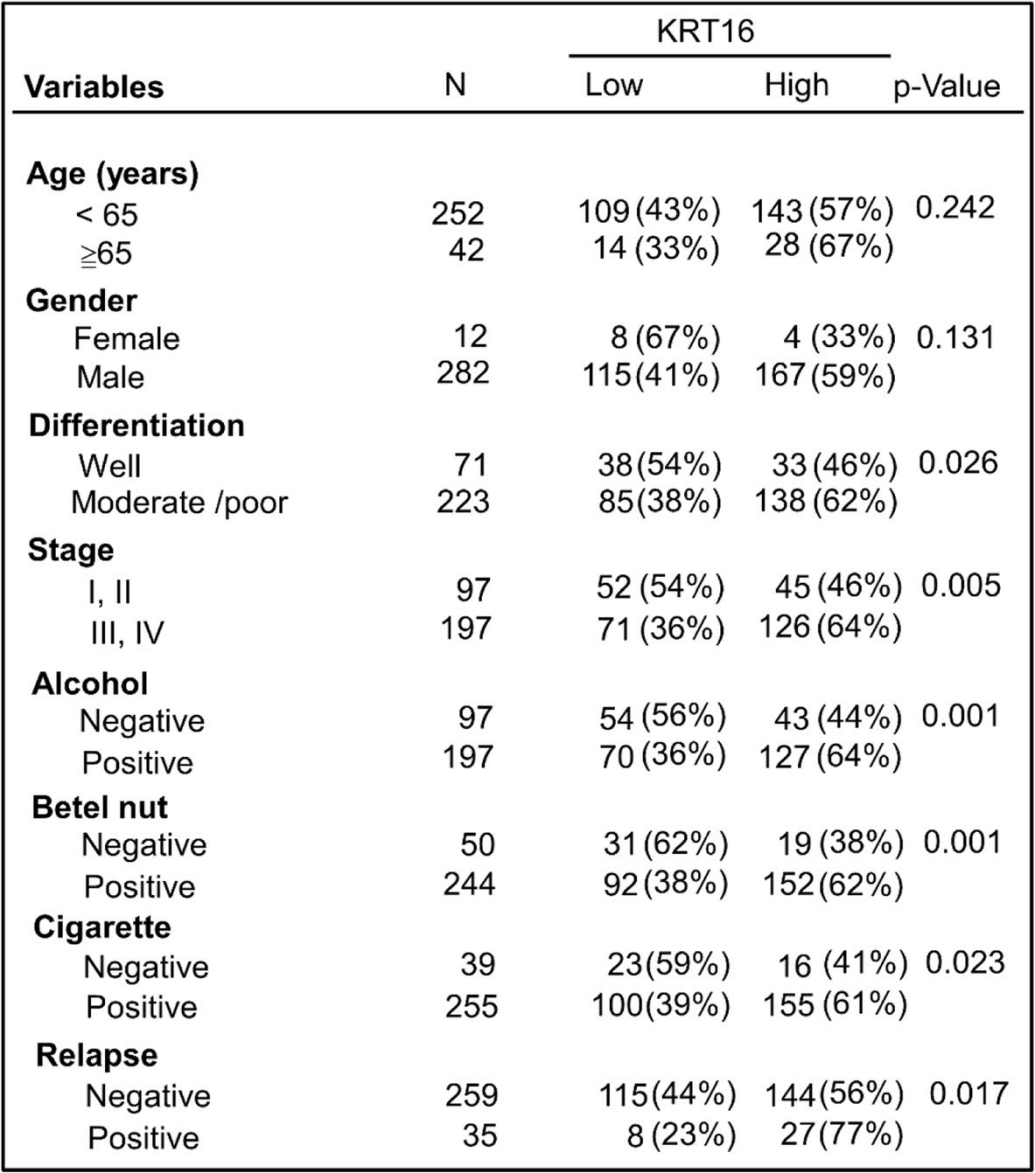
The relationship between clinical parameters and KRT16 expression in OSCC patients

### Depletion of KRT16 leads to decreased migration, invasion, metastasis, and cancer stemness in OSCC cells

**Fig. 2:**
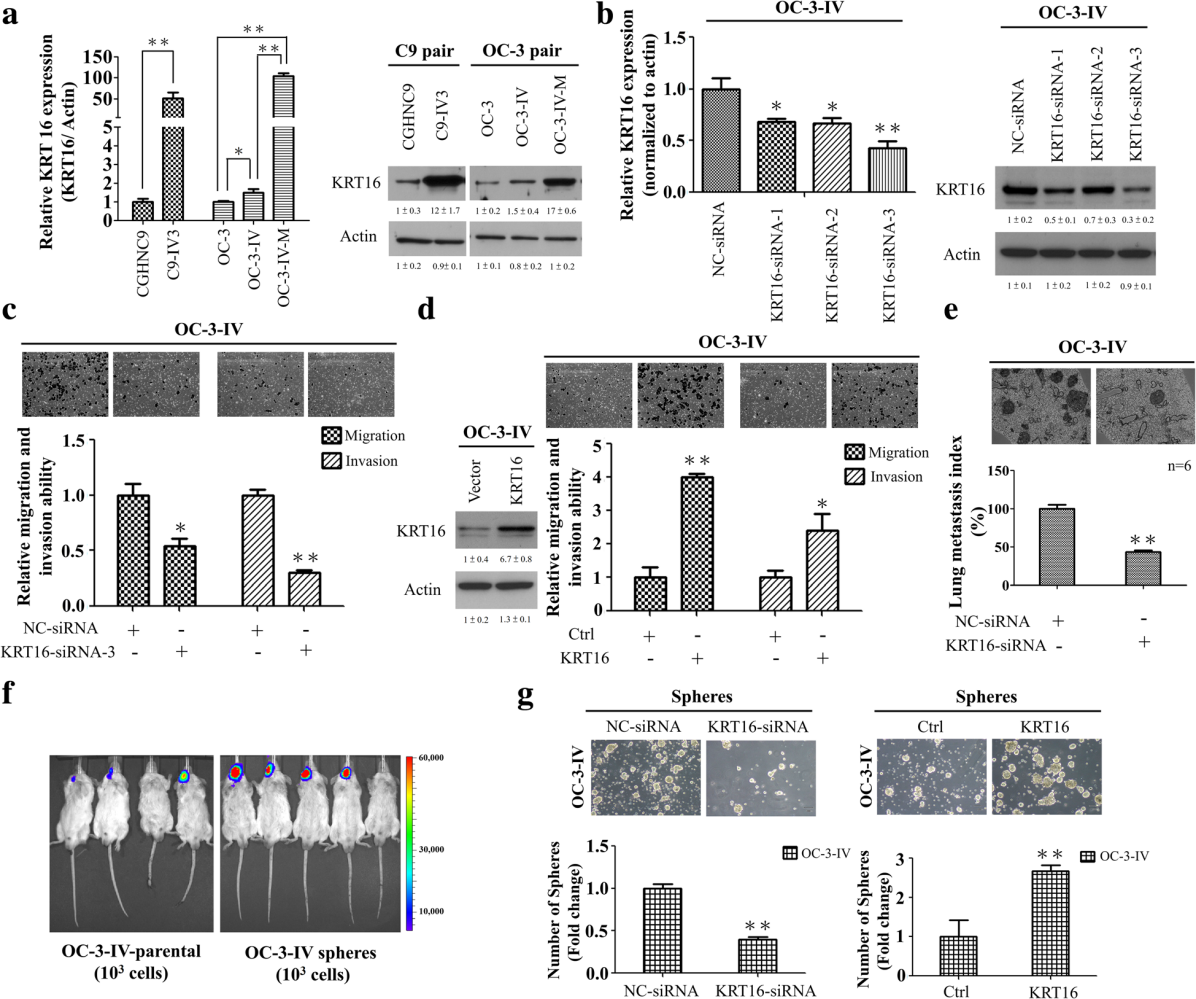
KRT16 regulates migration, invasion, sphere formation, and the metastatic abilities of OSCC cells. **a** The expression levels of KRT16 in two series of in vivo selected highly invasive lines (C9 pair and OC-3 pair) were measured through qRT-PCR (left) and immunoblotting (right). The actin was used as an internal control. **b** Left, the inhibitory efficacy of three specific KRT16-siRNAs. Right, immunoblotting of KRT16 protein in OC-3-IV cells transfected with the KRT16-siRNAs or NC-siRNA. **c** A decrease in migration and invasion abilities was observed in OC-3-IV cells transfected with KRT16-siRNA-3 compared with the control (NC-siRNA). **d** Left, immunoblotting of KRT16 from OC-3-IV cells transfected with the KRT16 plasmid or control vector. Right, cell migration and invasion were increased through ectopic expression of KRT16 in OC-3-IV cells. **e** Lung metastasis was reduced following tail vein injection of 1 × 10^6^ OC-3-IV cells transiently transfected with KRT16-siRNA. **f** Representative xenograft tumors formed by 1 × 10^3^ OC-3-IV sphere cells in the CB17-SCID mice. Tumor growth was monitored through BLI. Representative BLI results are shown at day 40 after implantation. **g** Sphere-forming ability of OC-3-IV cells was suppressed by KRT16-siRNA and enhanced by KRT16 overexpression. Sphere formation under stem cell selective condition was examined on day 10 after culturing of the cells transfected with the indicated siRNA or KRT16-plasmid. The original magnification was 40x. Histograms represent means ± SD from three independent experiments (*P < 0.05, **P < 0.01)

KRT16 is typically and strongly expressed in squamous cell carcinomas of different sites [20]. Our data showed that the KRT16 expression level was higher in highly invasive lines and OSCC tissues (Fig. 1a, b and e). Thus, we further explored possible functional roles of KRT16 in OSCC cells. Increase of KRT16 mRNA and protein expression level was found in three in vivo selected highly invasive lines, C9-IV3, OC-3-IV and OC-3-IV-M, compared with their parental CGHNC9 or OC-3 sublines respectively (Fig. 2a). Microarray analysis showed that the expression of KRT16 increased by 1.31 fold and 8.65 fold in OC-3-IV and OC-3-IV-M sublines, respectively, when compared with the parental OC-3 cells (Fig. 1a). Three specific KRT16-siRNAs were tested for their inhibitory efficacy by analyzing the KRT16 mRNA and protein levels. KRT16-siRNA-3 had the highest knockdown effect in inhibiting both KRT16 mRNA and protein (Fig. 2b), and it was used in the subsequent experiments. Knockdown of KRT16 significantly inhibited migration and invasion (Fig. 2c, Additional file 1: Figure S5a), whereas ectopic expression of KRT16 enhanced migration and invasion in OC-3-IV and CGHNC9 lines (Fig. 2d, Additional file 1: Figure S5a). To evaluate whether KRT16 promotes cancer cell metastasis in vivo, we employed an experimental metastasis model via tail vein injection of OSCC cells in CB17-SCID mice. In this model, knockdown of KRT16 significantly suppressed lung metastasis by approximately 60% compared with the control in OC-3-IV cells (Fig. 2e). Furthermore, we established OC-3-IV- and OC-3-IV-M-pSuper-puro cells stably expressing shKRT16 or empty vector (Additional file 1: Figure S1a). Indeed, analysis of these two shKRT16 stable lines (OC-3-IV- and OC-3-IV-M-shKRT16 cells) showed that depletion of KRT16 by stable shKRT16 resulted in decreased migration, invasion and metastasis abilities, and upon ectopic expression of KRT16 rescued the KRT16 depletion-mediated inhibition of migration, invasion and lung metastasis by approximately 60–80% (Additional file 1: Figure S5b and c).

To investigate whether KRT16 could affect cancer stemness of OSCC, we enriched the oral cancer stem-like cells from both OC-3-IV and C9-IV3 sublines by sphere-forming assay (Additional file 1: Figure S6a). These sphere-dispersed (sphere cells) cells had elevated mRNA levels of important cancer stem cell (CSC) makers including CD44, CD133, ALDH, ABCG2, OCT4, and Nanog compared with their parental cells in both OC-3-IV and C9-IV3 sublines (Additional file 1: Figure S6b). To investigate the tumorigenic potential of these sphere cells, 10^3^ cells of the OC-3-IV parental or sphere cells were implanted into CB17-SCID mice at the oral mucosa. Tumor growth was more prominent after implantation of sphere cells compared with parental cells with a tumor latency of 40 days indicating potent tumorigenicity of these cancer stem-like cells (Fig. 2f). Depletion of KRT16 caused a significant decrease in sphere formation, whereas overexpression of KRT16 increased it markedly (Fig. 2g). Furthermore, depletion of KRT16 by stable shKRT16 resulted in decreased sphere formation in OC-3-IV and OC-3-IV-M cells (Additional file 1: Figure S6c). Taken together, depletion of KRT16 impaired migration, invasion, metastasis, and cancer stemness of OSCC cells.

### EHF binds to the promoter region of the KRT16 gene and drives its transcriptional activity

**Fig. 3:**
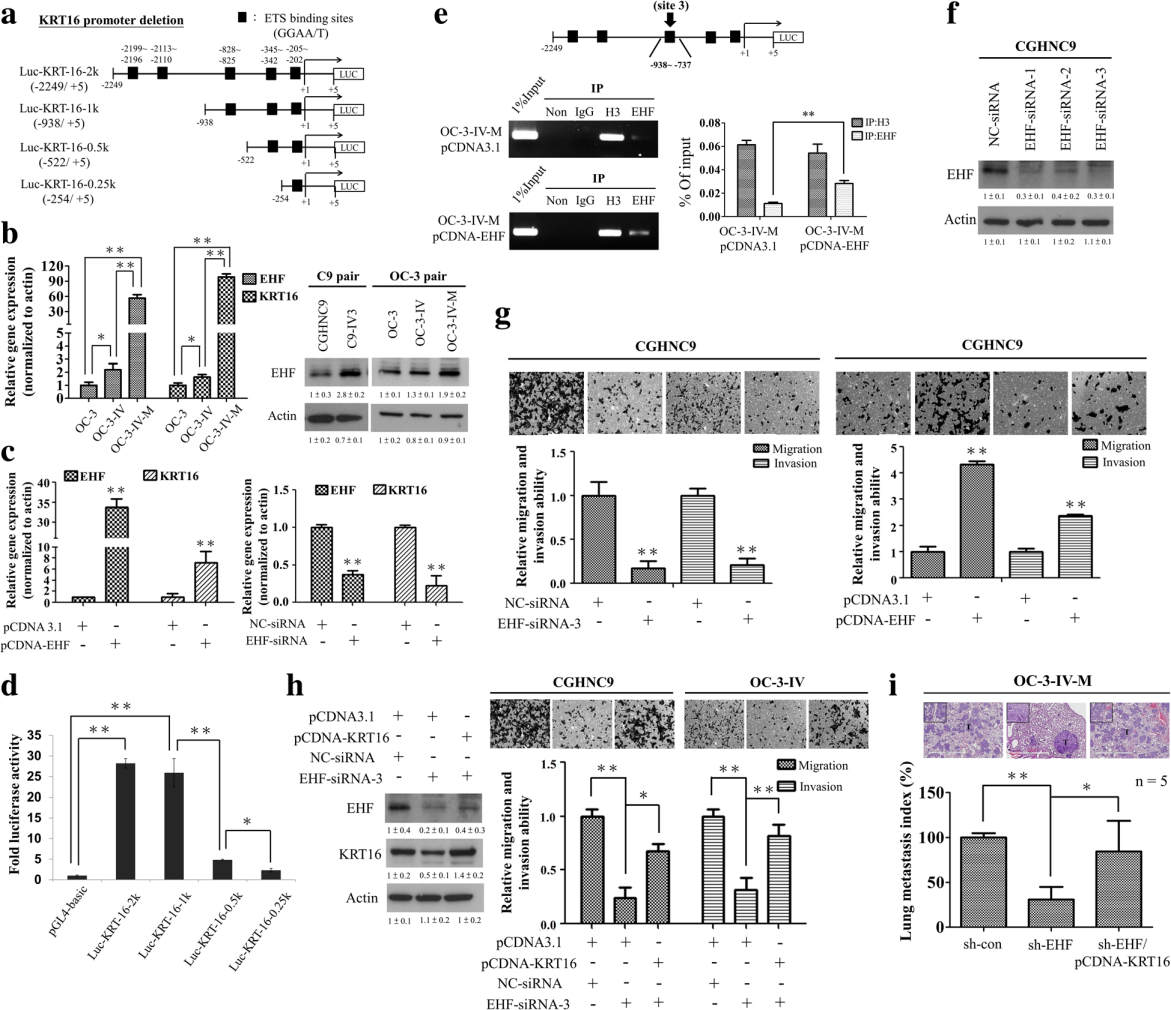
EHF promotes cell migration, invasion, and metastasis through the upregulation of KRT16 in OSCC cells. **a** Plasmid constructs for the KRT16 promoter–driven luciferase reporter assays including wild-type (KRT16–2249 ~ + 5) and three deletion mutants of ETS binding sites depicted by black boxes. **b** Left, the expression levels of EHF and KRT16 mRNAs in OC-3 pair were measured using qRT-PCR (*P < 0.05, **P < 0.01). Right, the expression levels of EHF protein in C9 pair and OC-3 pair were analyzed through immunoblotting. **c** Effect of overexpression (left) or knockdown (right) of EHF on endogenous KRT16 mRNA expression in OC-3-IV cells. **d** The wild-type and mutant KRT16 promoter–driven expressions of luciferase. **e** OC-3-IV cells were transfected with pcDNA3.1 (control) or EHF plasmid, and the ChIP assay was performed using primers specific for the site-3 region. The PCR product was analysed with agarose gel electrophoresis (Left) or qRT-PCR (Right). Histone 3 (H3) was used as a positive control. **f** Immunoblotting of EHF protein in CGHNC9 cells transfected with the EHF-siRNAs or NC-siRNA. **g** Left, the migration and invasion ability was decreased in CGHNC9 cells transfected with EHF-siRNA-3 compared with the control. Right, cell migration and invasion were increased through ectopic expression of EHF in CGHNC9 cells. **h** Left, the expression levels of EHF and KRT16 protein in OC-3-IV cells were analyzed through immunoblotting. Right, ectopic expression of KRT16 significantly restored the inhibition of migration and invasion due to EHF knockdown. **i** Lung metastasis of OC-3-IV-M-shEHF stable cells generated by 1 × 10^6^ cells injected into the tail vein of CB17-SCID mice was inhibited by EHF downregulation and was significantly restored by transfection with KRT16. The respective images display H&E staining of the lung metastases (200x) of each treatment group. Histograms in **(b), (c), (d), (e), (g), (h)** and **(i)** represent means ±SD from three independent experiments (*P < 0.05, **P < 0.01). The actin was used as an internal control

To elucidate the upstream regulation of KRT16, we sought putative transcriptional factor binding sites in the KRT16 promoter (up to 2.2 kb upstream of the transcriptional start site) using the Genomatix software. We identified five consensus transcriptional factor ETS binding sites (consensus = 5’GGAA/T3’) that were located in the KRT16 promoter (Fig. 3a, black boxes). We found that mRNA expression levels of both EHF and KRT16 were higher in the highly invasive OC-3-IV-M subline compared to parental OC-3 and OC-3-IV sublines (Fig. 3b, left). C9-IV3 cell line also gave similar results in mRNA expression levels of both EHF and KRT16 (Additional file 1: Figure S7a). The protein expression levels of EHF also increased in the two in vivo selected highly invasive lines, C9-IV3 and OC-3-IV-M, compared with their parental lines (Fig. 3b, right). Transfection with the expression vector of EHF strongly promoted KRT16 mRNA expression (Fig. 3c, left), whereas EHF knockdown resulted in significantly decreased KRT16 mRNA expression (Fig. 3c, right). To further explore the transcriptional regulatory role of EHF on KRT16, we cloned the human KRT16 promoter (− 2249/+ 5 bp) into a luciferase reporter plasmid (pGL3-Basic) and generated a series of truncated constructs (Fig. 3a). As shown in Fig. 3d, the deletion analysis of the KRT16 promoter revealed that full-length (Luc-KRT-16-2 k) and − 938 to + 5 (Luc-KRT-16-1 k) constructs were associated with a 25- to 28-fold increase in luciferase activities. However, an approximately 5- to 6-fold decrease in the luciferase activity of Luc-KRT-16-0.5 k compared with that of Luc-KRT-16-1 k was observed (Fig. 3d), suggesting that the region between − 938 and − 522 bp contained one or more positive regulatory elements. Potential EHF binding site 3 (site 3: − 828/− 825) is located in this region. Therefore, we designed primers spanning this GGAA/T site’s positions for ChIP assay. The results showed that the EHF overexpression (EHF plasmid) group had a significant ChIP signal corresponding to site 3 compared with the control (pCDNA3.1) group in OC-3-IV-M cells (Fig. 3e). These results indicated that the promoter region containing EHF binding site 3 was at least partly responsible for the positive regulation of the KRT16 gene transcription.

### Depletion of EHF impairs migration, invasion, and metastasis through inhibition of KRT16

To investigate the functions affected by EHF, CGHNC9 cells were transfected with three siRNAs targeting different coding regions of EHF and all of the three siRNAs effectively suppressed EHF mRNA (Additional file 1: Figure S7b) and protein levels (Fig. 3f). EHF-siRNA-3 was used in the subsequent experiments and was found to significantly inhibit the migration and invasion in CGHNC9 cells (Fig. 3g, left), whereas ectopic expression of EHF consistently enhanced their migration and invasion (Fig. 3g, right). Knockdown of EHF led to decrease of the KRT16 protein level, which could be partially rescued by transfecting KRT16 plasmid (Fig. 3h, left). Ectopic expression of KRT16 reversed EHF depletion-mediated inhibition of migration and invasion abilities (Fig. 3h, right). Furthermore, we established OC-3-IV- and OC-3-IV-M-pSuper-puro cells stably expressing shEHF or empty vector (Additional file 1: Figure S1b). Depletion of EHF by expressing shEHF resulted in significantly decreased migration and invasion abilities in OC-3-IV and OC-3-IV-M lines, and ectopic expression of KRT16 was able to significantly rescue shEHF-mediated inhibition of migration and invasion in OSCC (Additional file 1: Figure S8). By tail vein injection assay, we found that depletion of EHF by stable shEHF expression decreased lung metastasis whereas ectopic expression of KRT16 reversed EHF depletion-mediated inhibition of lung metastasis by approximately 80% in the OC-3-IV-M line (Fig. 3i). These results supported that depletion of EHF impaired migration, invasion, and metastasis through inhibition of KRT16 in OSCC.

### MiR-365-3p targets EHF to inhibit OSCC migration, invasion, and metastasis through KRT16

Reports have revealed that miRNAs affect different steps of cancer tumorigenesis [21]. To explored the upstream regulators of EHF in OSCC cells, we attempted to identify potential miRNAs that might be involved in targeting EHF 3’UTR. Using TargetScan software analysis, four conserved miRNAs were identified and their expression levels were investigated in OC-3-IV and OC-3-IV-M sublines by qRT-PCR. Among the four miRNAs, the expression of both miR-365-3p and miR-505-3p significantly downregulated in the OC-3-IV-M subline (Additional file 1: Figure S9a). However, only miR-365-3p was found to be able to inhibit the EHF mRNA expression (Additional file 1: Figure S9b).

**Fig. 4:**
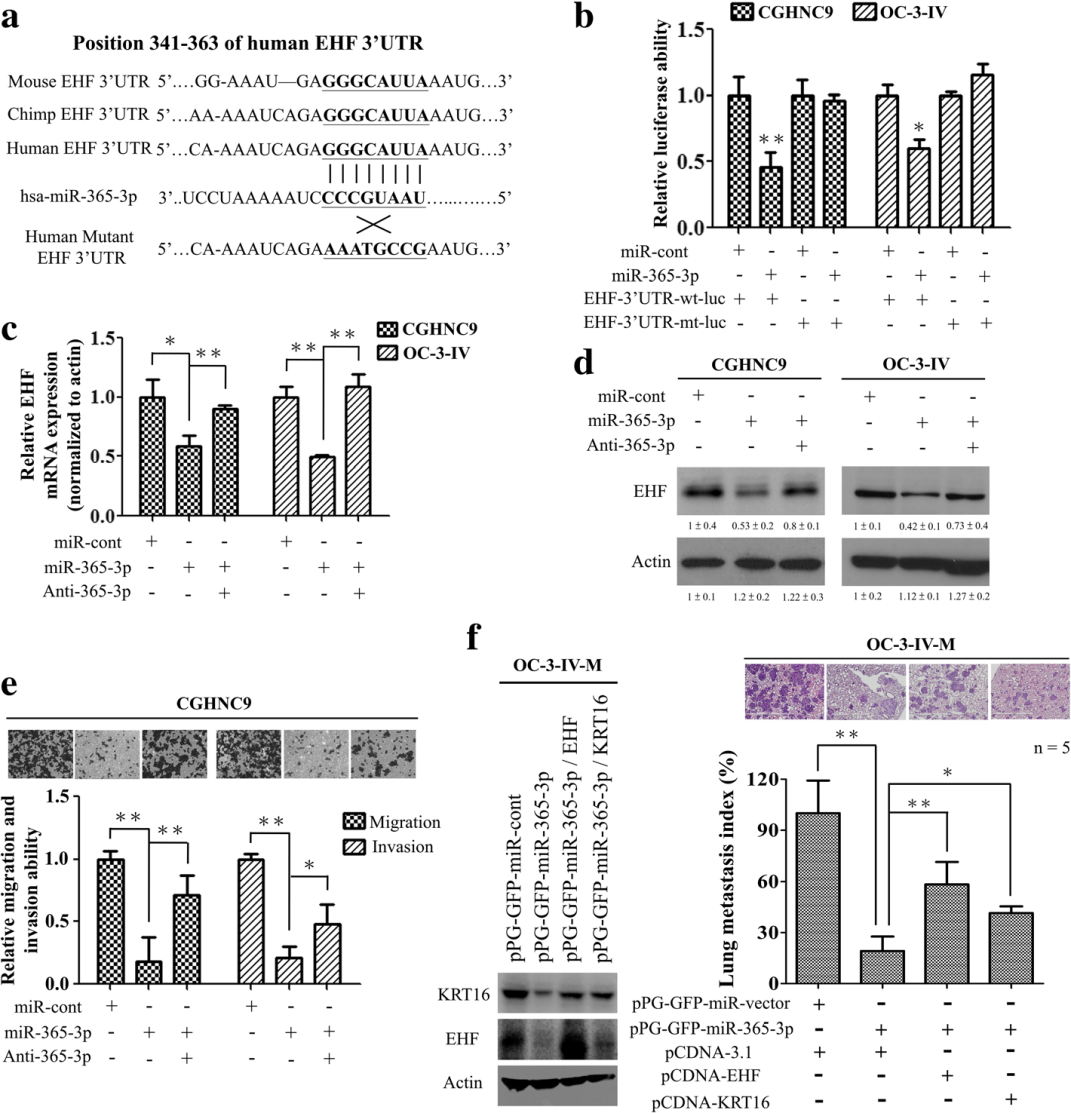
EHF is a direct target of miR-365-3p in OSCC cells. **a** Schematics of the highly conserved miR-365-3p binding site in human EHF 3’-UTRs. The miR-365-3p binding sequences and the mutant 3’UTR binding site are shown. **b** Luciferase activity driven by the wild-type (wt) and the mutant (mt) EHF 3’-UTR reporter constructs in the presence of miR-365-3p. **c** and **(d)** The effects of pre-miR-365-3p transfection on EHF mRNA and protein expression in CGHNC9 and OC-3-IV cells were analyzed by qRT-PCR and immunoblotting respectively. The actin was used as an internal control. **e** Transient transfection of miR-365-3p significantly inhibited migration and invasion of CGHNC9 cells, which was reversed by anti-miR-365-3p. **f** Left, the protein expression levels as reflected by Western blotting of EHF and KRT16 in OC-3-IV-M stable cells transfected with the indicated plasmids are shown. Right, lung metastasis via tail vein injection of 1 × 10^6^ OC-3-IV-M cells into CB17-SCID mice was significantly inhibited in OC-3-IV-M-miR-365-3p stable cells and was restored by transfection with EHF or KRT16. Histograms in **(b), (c), (e)** and (**f)** represent means ± SD from 3 independent experiments (*, P < 0.05; **, P < 0.01)

To test whether EHF is a direct target of miR-365-3p, a luciferase reporter plasmid containing the EHF 3’UTR harboring a conserved miR-365-3p binding site was constructed (Fig. 4a). The reporter assay revealed that the miR-365-3p significantly reduced the EHF promoter driven luciferase activity in the OSCC cells and the mutation of the miR-365-3p binding site abolished the inhibition by miR-365-3p (Fig. 4b). Subsequently, we demonstrated that transfection of miR-365-3p reduced the endogenous EHF mRNA and protein expression in OSCC cells, and this inhibition could be reverted by cotransfection with anti-miR-365-3p (Fig. 4c and d).

To further assess the functional roles of miR-365-3p in regulating EHF, we found that overexpression of miR-365-3p significantly inhibited migration and invasion in OSCC cells, and the inhibition was significantly reverted by cotransfection with anti-miR-365-3p (Fig. 4e). We also established OC-3-IV- and OC-3-IV-M-pPG-GFP-miR cells stably expressing miR-365-3p or empty vector tagged with green fluorescent protein (GFP) gene (Additional file 1: Figure S1c) and confirmed that ectopic expression of EHF or KRT16 reversed miR365-3p-mediated inhibition of migration and invasion (Additional file 1: Figure S10). In metastasis assay, OC-3-IV-M cells expressing miR-365-3p or empty vector were injected into CB17-SCID mice through tail veins. The result showed that overexpression of miR-365-3p lead to significant decrease of lung metastasis, whereas ectopic expression of EHF or KRT16 effectively restored the miR-365-3p–mediated inhibition (Fig. 4f). These results indicated that miR-365-3p targets EHF to inhibit OSCC migration, invasion, and metastasis through KRT16.

### Depletion of KRT16 leads to the enhanced lysosomal degradation of β5-integrin and c-met in OSCC cells

**Fig. 5:**
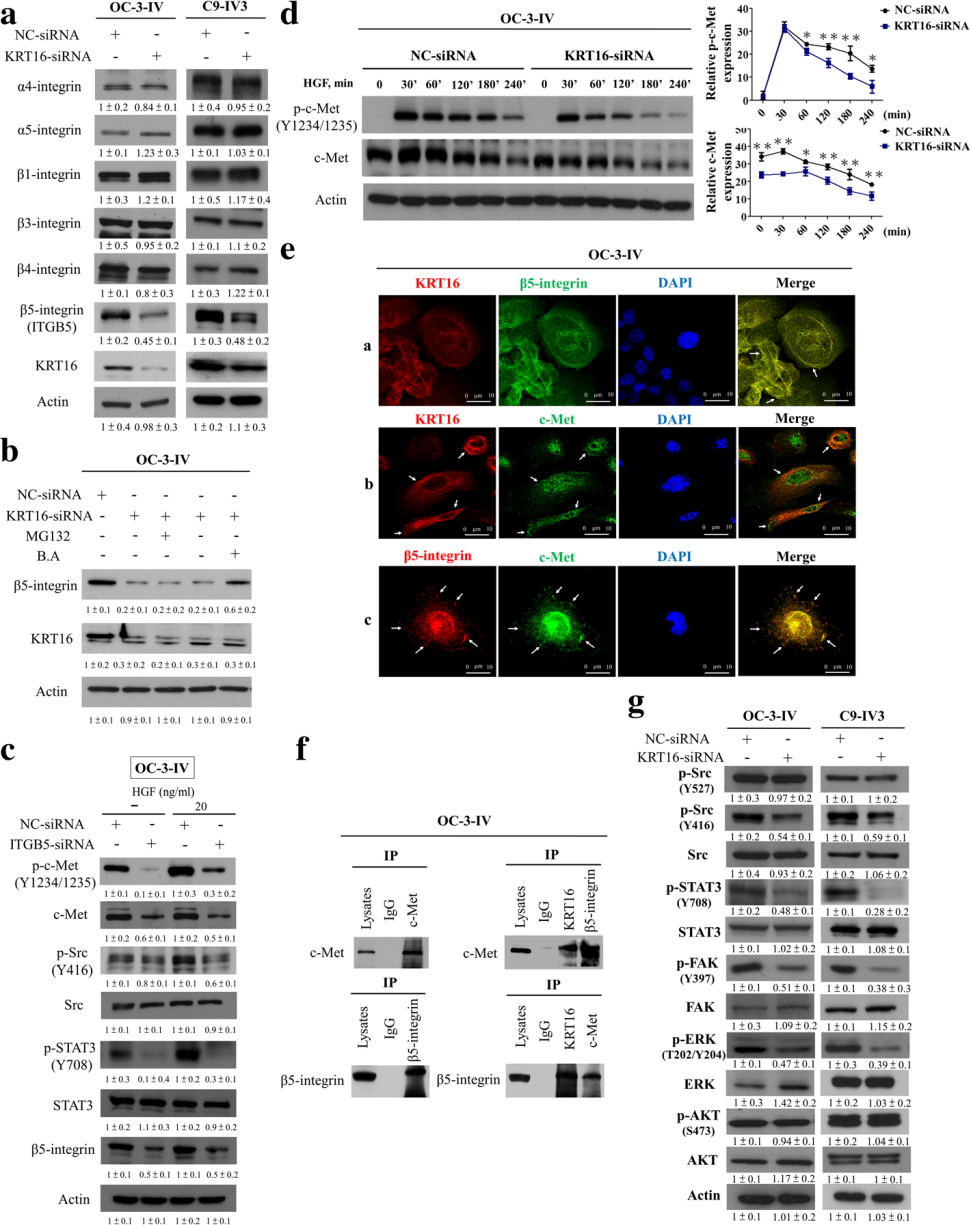
KRT16 stabilizes β5-integrin and c-Met to transmit downstream signaling through Src/STAT3 signaling pathway. **a** Analysis of integrin isoforms in KRT16-siRNA–transfected OC-3-IV and C9-IV3 cells. **b** Treatment with 10 μM MG132 for 8 h could not prevent the loss of β5-integrin (ITGB5) in KRT16-depleted cells. Treatment with 100 nM bafilomycin A (B.A.) for 24 h prevented ITGB5 degradation in KRT16-depleted cells. **c** NC-siRNA and β5-integrin-silenced OC-3-IV cells were serum starved for 18 h O/N, treated with 20 ng/mL HGF for 30 min, and subjected to immunoblotting. Depletion of KRT16 depletion enhanced degradation of c-Met in OC-3-IV cells. Knockdown of KRT16 decreased c-Met protein, which was blocked by B.A. rather than MG132. **d** Left, OC-3-IV cells were transfected with the indicated plasmids for 8 h, serum starved for 24 h, and then were treated with 50 ng/mL HGF for the indicated times. Right, the p-c-Met and c-Met levels were shown and quantified. **e** Localization of KRT16, c-Met, and β5-integrin was analyzed in OC-3-IV cells through confocal microscopy. Panel a, IF staining under confocal microscopy of KRT16 (red), β5-integrin (green), and nuclei (DAPI, blue) after adhesion of OC-3-IV cells to Poly-L-Lysine; panel b, IF staining under confocal microscopy of KRT16 (red), c-Met (green), and nuclei (DAPI, blue); panel c, IF staining under confocal microscopy of β5-integrin (red), c-Met (green), and nuclei (DAPI, blue). **f** Cell lysates were blotted directly or subjected to IP with the indicated antibodies followed by blotting with the indicated antibodies. IgG served as the negative control. **g** Effect of KRT16 knockdown on c-Met signaling-related molecules in OC-3-IV and C9-IV3 cells. All the in vitro experiments were performed in triplicates and repeated three times. The actin was used as an internal control

We subsequently explored the mechanism of the KRT16-mediated regulation of invasion, migration, and metastasis in OSCC cells. β4-integrin has been reported to interact with keratin filaments through plectin, and the absence of keratins leads to loss of plectin-β4-integrin complex formation [16]. To determine if KRT16 promotes tumor progression by regulating integrins and their signaling, we examined the expression level of integrin isoforms in KRT16-depleted OSCC cells. Immunoblotting analysis revealed that depletion of KRT16 led to a significantly decreased level of β5-integrin in three OSCC lines (OC-3-IV, OC-3-IV-M and C9-IV3) and OC-3-IV-M-shKRT16 stable cells (Fig. 5a and Additional file 1: Figure S11a). We further examined whether the reduction of β5-integrin was due to enhanced proteasomal or lysosomal degradation in these three OSCC lines (OC-3-IV, OC-3-IV-M and C9-IV3). The β5-integrin levels in KRT16-depleted cells with or without MG132 (a proteasome inhibitor) or B.A. (a lysosome inhibitor) treatment were compared. The results showed that only B.A. treatment rescued the β5-integrin level owing to KRT16 depletion (Fig. 5b and Additional file 1: Figure S11b), suggesting that KRT16 knockdown increased β5-integrin degradation through the lysosomal pathway in OSCC cells. To further confirm if depletion of KRT16 could affect the stability of β5-integrin, we conducted protein degradation experiment in cells treated with cycloheximide (Cyclohex). Β5-integrin was stable over a course of 12 h in the control cells whereas the stability of β5-integrin was significantly decreased in the KRT16-depleted cells (Additional file 1: Figure S11c).

A study indicated that silencing β4-integrin suppressed c-Met signaling in human prostate cancer cells [22]. Moreover, expression of high levels of integrins or c-Met is correlated with disease progression in various tumor types [23]. Our results also showed that increase of c-Met protein expression level was observed in the two highly invasive lines, especially for OC-3-IV-M, compared with the parental line, OC-3 (Additional file 1: Figure S12a). Furthermore, our data reveals that knockdown of β5-integrin led to reduction of c-Met protein level, and suppressed HGF-induced phosphorylation of c-Met as well as its downstream signaling mediators, Src and STAT3, in OSCC cells (Fig. 5c and Additional file 1: Figure S12b). In addition, we also examined whether KRT16 affected c-Met only or affected other receptor tyrosine kinases (RTKs) as well. The results revealed that inhibiting KRT16 expression only reduced the c-Met protein level and its phosphorylation among the RTKs analyzed (Additional file 1: Figure S12c). We also demonstrated that depletion of endogenous KRT16 could reduce c-Met level, and B.A. treatment reversed the effect of KRT16 knockdown (Additional file 1: Figure S11b and d). This suggests that KRT16 knockdown induced c-Met degradation is through the lysosomal pathway.

Next, by depletion of KRT16 and subsequent stimulation with hepatocyte growth factor (HGF) in OC-3-IV, OC-3-IV-M and C9-IV3 cells, we found that both c-Met and its phosphorylation were significantly decreased in KRT16-depleted cells (Fig. 5d and Additional file 1: Figure S12d). We further examined whether miR-365-3p or EHF-siRNA resulted in significant decrease of c-Met expression, we found that miR-365-3p or EHF-siRNA significantly reduced c-Met protein level in three OSCC lines (OC-3-IV, OC-3-IV-M and C9-IV3) (Additional file 1: Figure S12e). Thus, these results suggest that KRT16 protects the c-Met protein form degradation leading to its stabilization and prolonged activation in OSCC cells, whereas suppression of KRT16 leads to disruption of β5-integrin/c-Met signaling through the lysosomal degradation of both proteins.

### KRT16 constitutively associates with β5-integrin and c-met to transmit downstream signaling through Src/STAT3 in OSCC cells

To further clarify the interactions among KRT16, β5-integrin, and c-Met, we examined the localization correlation of these three components in three OSCC cells by IF. Through confocal microscopic examination, KRT16 and β5-integrin (Fig. 5e and Additional file 1: Figure S13a, panel a), as well as β5-integrin and c-Met (Fig. 5e and Additional file 1: Figure S13a, panel c) displayed similar spatial pattern and were colocalized at the cell surface and protrusions. However, KRT16 and c-Met (Fig. 5e and Additional file 1: Figure S13a, panel b) only partially overlapped at the cell surface. Interestingly, c-Met was frequently detected near KRT16. Our observations suggested that c-Met possibly partially associates with KRT16 through β5-integrin and these three proteins may colocalize to form stabilized complexes to facilitate c-Met and β5-integrin–mediated signaling in OSCC cells. To test the hypothesis, we conducted co-IP assays among KRT16, β5-integrin, and c-Met in three OSCC lines. Our reciprocal co-IP experiments revealed association among KRT16, β5-integrin, and c-Met proteins (Fig. 5f, Additional file 1: Figure S13b and c). To test whether the anti-KRT16 antibody cross reacted with KRT14 in the Co-IP experiments, KRT14 recombinant protein (2 μg) (Protein Specialists, inc.) was added to cell lysates to compete for binding to the antibody. Our data showed that the immunoblotting pattern for KRT16 showed no significant change with or without KRT14 recombinant protein competition (Additional file 1: Figure S13d). We also conducted Kaplan–Meier survival analysis of 56 cases in a Taiwanese cohort series and revealed that patients with OSCC with higher simultaneous expression levels of these three genes (KRT16, β5-integrin, and c-Met) had significantly poorer clinical survival than those with lower expression levels of the three genes, similar results were also found in individual gene expression (Additional file 1: Figure S14).

A previous study indicated that β1-integrin promotes the endocytosis of activated c-Met as well as c-Met signaling post-endocytosis on autophagy-related endomembranes [24]. Microtubule-associated protein 1A/1B-light chain 3 (LC3) is known to be associated with the autophagic pathway and could be attributed to either the induction of early stages of autophagy or the inhibition of late stages of autophagic flux [15, 25]. Since our data suggested that KRT16, β5-integrin and c-Met might form a stable protein complex, we further investigated whether autophagic process was involved in the regulation of c-Met endocytosis. By Western blotting analysis, we found that depletion of KRT16 led to a decrease of c-Met and an increase of LC3 protein level in KRT16 siRNA-transfected OC-3-IV-M and C9-IV3 cells after 8 h of transfection (Additional file 1: Figure S15a). Immunofluorescence staining showed similar results that LC3 was increased in depleted KRT16 OC-3-IV-M cells (Additional file 1: Figure S15b). We also found that the internalized c-Met were more abundantly co-localized with LC3 in the KRT16 depleted cells compared with control cells (Additional file 1: Figure S15b). These results implied that KRT16 depletion led to autophagy activation, which also contributed to increase endocytosis of c-Met.

A previous report revealed that binding of HGF to c-Met triggers receptor homodimerization leading to subsequent promotion of Src/STAT3 signaling and cancer progression [26]. Therefore, we examined c-Met and integrin-mediated signaling pathways including the Src/STAT3 and the FAK/ERK axis. Immunoblotting revealed that the active form of Src (p-Src-416) and phosphorylation of STAT3, FAK and ERK were reduced in the KRT16-depleted cells (Fig. 5g and Additional file 1: Figure S16a). However, KRT16 suppression did not reduce AKT activation in the OC-3-IV, OC-3-IV-M and C9-IV3 cells (Fig. 5g and Additional file 1: Figure S16a). Depletion of c-Met or β5-integrin reduced phosphorylation of Src and inhibited the migration and invasion of OSCC cells (Additional file 1: Figure S16b). To examine if the activation of c-Met, STAT3, and Src contributed to the migration and invasion of OSCC highly invasive cells, we treated with the c-Met inhibitor (PHA-665752), Src inhibitor (SRCIN1) and STAT3 inhibitor (WP1066). Our data showed that treatment with c-Met, Src and STAT3 inhibitor significantly inhibited cell migration and invasion (Additional file 1: Figure S16c and d). In addition, we found that treatment with STAT3 inhibitor (WP1066) suppressed cell proliferation at least on the third day whereas treatment with c-Met and Src inhibitors (PHA-665752 and SRCIN1) had no significant suppression effect on cell proliferation compared to the control (Additional file 1: Figure S16e).

STAT3 is also known to be a downstream of Janus kinase 2 (JAK2) [27]. The JAK2/STAT3 signaling mediates the effects of many growth factors and cytokines, and has been intensely investigated in many cancer types [28]. To determine whether KRT16 trigged the STAT3 signaling via JAK2 or Src activation, we treated with Src inhibitor and JAK2 inhibitor in KRT16 overexpressing OC-3-IV-M cells. Our data showed that treatment with Src inhibitor significantly suppressed phosphorylation of STAT3 in KRT16 overexpressing OC-3-IV-M cells, whereas inhibition of JAK2 via JAK2 inhibitor had no effect on STAT3 activation in KRT16 overexpressing OC-3-IV-M cells (Additional file 1: Figure S17). These results indicate that Src is mainly responsible for phosphorylation of STAT3 in KRT16 overexpressing OC-3-IV-M cells.

Taken together, our data suggest that KRT16 stabilizes β5-integrin and c-Met leading to enhanced downstream signaling of Src/STAT3 pathway to promote OSCC cells migration, invasion and metastasis.

### Targeting miR-365-3p/EHF/KRT16/β5-integrin/c-met signaling axis increases the cytotoxic effect of 5-FU in the treatment of OSCC

**Fig. 6:**
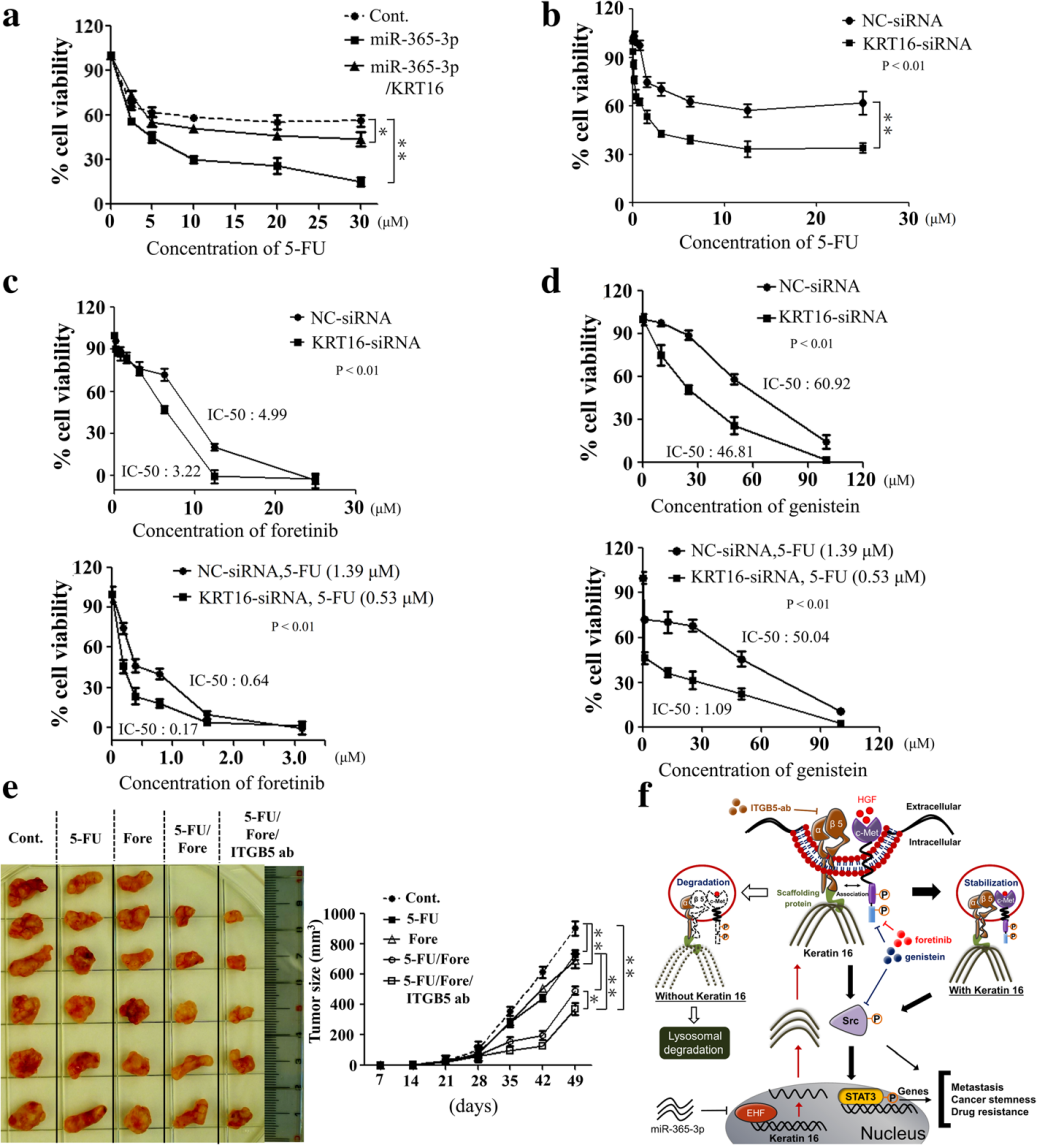
Inhibition of miR-365-3p/KRT16/β5-integrin/c-Met signaling pathway in OSCC cells enhances chemosensitivity towards 5-FU treatment. **a** OC-3-IV cells were transfected with indicated plasmids for 48 h, and then were treated with various concentrations of 5-FU continuously for an additional 18 h. MTT assay was conducted to measure cell growth after 72 h. **b** Dose-dependent growth inhibitory effect on OC-3-IV cells or KRT16-depleted OC-3-IV cells upon continuous exposure to 5-FU as indicated concentration through MTT assay. **c** Top, dose-dependent growth inhibitory effect in OC-3-IV cells or KRT16-depleted OC-3-IV cells after continuous exposure to foretinib (a c-Met inhibitor) as indicated concentration through MTT assay. Bottom, KRT16 knockdown further sensitized OC-3-IV cells toward combined 5-FU and foretinib treatment. **d** Top, dose-dependent growth inhibitory effect in OC-3-IV cells or KRT16-depleted OC-3-IV cells after continuous exposure to genistein as indicated concentration through MTT assay. Bottom, KRT16 knockdown further sensitized OC-3-IV cells toward combined 5-FU and genistein treatment. **e** Combination treatment of 5-FU, foretinib, and ITGB5-ab markedly reduced tumor size in vivo. Left, CB17-SCID mice were categorized into five groups according to the treatment of compounds as indicated. In each group, 1 × 10^6^ OC-3-IV cells were subcutaneously implanted into CB17-SCID mice separately. Right, tumor growth was monitored. All mice were sacrificed on day 56 after implantation. All the in vitro experiments were performed in triplicates and repeated three times (*P < 0.05, **P < 0.01). **f** The model of a novel miR-365-3p/EHF/KRT16/β5-integrin/c-Met cascade signaling pathway. KRT16 is associated with β5-integrin and c-Met with stabilization leading to enhanced c-Met signaling as well as activation of Src/STAT3 signaling to promote metastasis, cancer stemness, and drug resistance in OSCC cells. The dotted lines represent the protein degradation

Although 5-FU is one of the main chemotherapeutic agents in treating OSCC, rapid development of chemoresistance remains an unsolved issue [29]. Effective therapeutic agents for OSCC are relatively scarce at present [30–32]. Evidence increasingly indicates that tumor suppressor miRNAs could be applied as treatment options [33]. To test if miR-365-3p affects chemoresistance in OSCC, OC-3-IV cells transfected with miR-365-3p resulted in significantly increased chemosensitivity towards 5-FU compared with control (Fig. 6a). Ectopic expression of KRT16 could partially restore chemoresistance towards 5-FU affected by miR-365-3p. These results indicated that miR-365-3p could be a potential strategy to target KRT16/β5-integrin/c-Met signaling.

We further assessed whether inhibition of the endogenous KRT16 could affect the chemosensitivity of OSCC. Initially we treated the OC-3-IV cells with escalating doses of 5-FU till a relatively high concentration of 25 μM, yet no IC_50_ level could be reached (Fig. 6b). This indicated that the OC-3-IV cell line was highly resistant to 5-FU and silencing KRT16 significantly enhanced chemosensitivity towards 5-FU (Fig. 6b). Besides, we also found that treatment with 5-FU significantly reduced the activation of c-Met/Src signaling, knockdown of KRT16 further enhanced the inhibitory effects (Additional file 1: Figure S18a). Upon treatment with foretinib, OC-3-IV cells were relatively chemosensitive (IC_50_ = 4.99 μM) compared to 5-FU treatment, and depletion of KRT16 augmented the chemosensitivity (IC_50_ = 3.22 μM) (Fig. 6c, top). For combination therapy with foretinib and 5-FU, we selected relative low doses of 5-FU, 1.39 μM or 0.53 μM, for NC-siRNA or KRT16-siRNA treated cells, respectively, to avoid unnecessary overly high cytotoxicity. The combination of foretinib with 5-FU led to a strong decrease in cell viability and much lower IC_50_ for foretinib (0.64 μM), and the IC_50_ was further reduced to 0.17 μM by simultaneous KRT16 knockdown (Fig. 6c, bottom). Thus, our data indicated that depletion of KRT16 significantly increased chemosensitivity of OSCC cells towards 5-FU and foretinib.

Genistein is a protein tyrosine kinase inhibitor to exert an inhibitory effect on various cancer cells by inducing G2/M arrest and apoptosis [34–37]. Our data revealed that treatment with genistein inhibited activation of c-Met/Src signaling in OSCC cells (Additional file 1: Figure S18b). Genistein also showed cytotoxic effects in the OC-3-IV line (IC_50_ = 60.92 μM) and depletion of KRT16 further increased their chemosensitivity (IC_50_ = 46.81 μM) (Fig. 6d, top). The combination of genistein with 5-FU led to further decrease in cell viability and lower IC_50_ for genistein (50.04 μM), depletion of KRT16 further reduced the IC_50_ to 1.09 μM (Fig. 6d, bottom).

We further examined the effect of KRT16 depletion in various combination treatments of 5-FU, foretinib, genistein, and ITGB5-ab for OSCC. Our results demonstrated that combination regimens with 5-FU, foretinib, and genistein increased cytotoxicity compared with single agent treatment, depletion of KRT16 further enhanced the cytototoxic effects in both OC-3-IV and C9-IV3 lines (Additional file 1: Figure S19a). Moreover, adding ITGB5-ab in combination with 5-FU/foretinib increased cytotoxic effects in the OC-3-IV line and depletion of KRT16 further augmented cytotoxicity (Additional file 1: Figure S19b). In animal model, we found that treatment with 5-FU or foretinib alone exhibited limited inhibitory efficacy of tumor growth in CB17-SCID mice (Fig. 6e). However, the combination with 5-FU and foretinib markedly inhibited tumor growth, which was further suppressed when combined with 5-FU, foretinib and ITGB5-ab (Fig. 6e). Taken together, our study unveiled a novel miR-365-3p/EHF/KRT16/β5-integrin/c-Met signaling axis regulating metastasis, cancer stemness, and drug resistance in OSCC (Fig. 6f).

## Discussion

CSCs have been identified with high potential to metastasize and often cause recurrence after treatment because of their resistance to chemotherapy and radiotherapy [14, 38]. In this study, we successfully enriched OSCC cancer stem-like cells from both OC-3-IV and C9-IV3 lines with important CSC characteristics. We also found that depletion of KRT16 lead to decreased migration, invasion, metastasis, and cancer stemness in OSCC cells. High KRT16 expression was found to be associated with more lymph node metastasis and poor clinical survival in our study. This indicated that KRT16 may serve as a novel prognostic and therapeutic target in OSCC treatment.

EHF is an ETS family transcription factor expressed in multiple epithelial cell types and plays a key role in regulating epithelial cell proliferation and differentiation [39, 40]. Knockdown of EHF inhibited proliferation, invasion, and tumorigenesis in ovarian cancers [41]. However, the biological roles of EHF in OSCC remain unknown. In this study, we are the first to demonstrate that miR-365-3p targeted EHF to inhibit OSCC cell migration, invasion, and metastasis through regulation of KRT16. Through targeting EHF, miR-365-3p could be considered as a candidate target for developing a novel therapeutic strategy for treating OSCC.

Integrins were found to regulate diverse cellular functions and play key roles in solid tumor progression and metastasis [15]. Studies have reported that integrins and plectins will not colocalize in the absence of keratins, leading to the loss of patchy hemidesmosomal assemblies and increased motility of the corresponding keratinocytes [12, 16]. Under confocal microscopic observation, we found that KRT16 and β5-integrin were colocalized, c-Met and β5-integrin were also colocalized. However, c-Met only partially associated with KRT16. Since c-Met is a transmembrane protein and can undergo endosomal internalization through HGF stimulation [42], HGF in our cell culture medium may facilitate c-Met endocytosis and some internalized c-Met is able to be associated with KRT16 through β5-integrin. In the other way, our data also implied that KRT16 depletion led to autophagy activation to promote the endocytosis of c-Met. The precise mechanisms involved in c-Met endocytosis and association with KRT16 through β5-integrin warrants further study. We also found that depletion of KRT16 increased β5-integrin and c-Met degradation through the lysosomal pathway and reduced invasion and metastasis in OSCC cells. Although the roles of numerous keratins have been identified as regulators of cancer progression in different cancers [5], our study was the first to document the interaction among KRT16, β5-integrin, and c-Met in cancer metastasis and the brand new role of KRT16 in stabilizing β5-integrin and c-Met as well as subsequent cascade signaling transduction in OSCC.

Previous reports indicated that dysregulation of the HGF/c-MET pathway was found in multiple tumor types and was associated with unfavorable outcomes [43]. Therefore, targeting the HGF/c-Met signaling has become a potential therapeutic strategy and several small-molecule c-Met kinase inhibitors have been generated and were in clinical trials since the early twenty-first century [43]. Some study showed that HGF-mediated c-Met internalization into clathrin-coated vesicles is under the control of protein kinase C and results in sustained signal attenuation [44–46]. Our finding demonstrated that the c-Met degradation and inactivation was enhanced upon KRT16 downregulation and β5-integrin inhibition is distinct from current approaches for inhibiting the HGF/c-Met signaling. From our results, KRT16 and β5-integrin protein could protect the internalization and degradation of c-Met receptor vesicles and could be alternative targets for c-Met inhibition.

Studies have indicated that integrins, in cooperation with RTKs, cause the activation of Src or other Src family kinases and recruit them to the peripheral signaling complexes [47, 48]. Some report indicated that the EGFR was associated with the hemidesmosomal α6β4-integrin in both normal and neoplastic keratinocytes [49]. Furthermore, β4-integrin was found to amplify ErbB2 and c-Met signaling pathways to promote prostate carcinoma cell proliferation and invasion [22]. These findings suggest that integrin signaling may promote tumor progression and metastasis through interaction with its scaffolding protein, keratin, and the RTK complex [22]. Our study demonstrated that KRT16 is constitutively linked with β5-integrin and c-Met, leading to activated Src/STAT3 rather than JAK2/STAT3 signaling as well as enhanced OSCC cell invasion and metastasis, and was in agreement with those previous findings.

Although 5-FU based chemotherapy was largely applied in treating OSCC, acquisition of chemoresistance was identified as the major problem preventing successful OSCC treatment [50]. Very few molecular targets except for EGFR have been proven as effective molecular targets for OSCC therapy to date [51]. In our study, we found that both 5-FU and genistein inhibited activation of c-Met/Src signaling in OSCC cells. Inhibition of KRT16 expression further enhanced 5-FU induced cytotoxicity through c-Met/Src signaling. Therefore, KRT16 could affect drug resistance through RTK signaling such as c-Met in OSCC. Our results also indicated that depletion of KRT16 could benefit the combination treatment of 5-FU, foretinib, genistein and ITGB5-ab for OSCC. These results indicate that the c-Met/Src signaling pathway could play a pivotal role in regulating the chemosensitivity of OSCC cells towards 5-FU.

## Conclusions

In conclusion, we discovered a novel regulatory cascade of miR-365-3p/EHF/KRT16/β5-integrin/c-Met signaling regulating oral cancer metastasis, cancer stemness, and drug resistance. We also found combined inhibitors of components of this signaling axis with 5-FU improved treatment efficacy in OSCC. Targeting this novel miR-365-3p/EHF/KRT16/β5-integrin/c-Met signaling pathway could be a promising strategy in the treatment of OSCC.

## Additional file

Additional file 1: **Figure S1.** Establishment of shKRT16, shEHF and miR-365-3p stably expressing cancer cell lines in invasive OSCC lines. **Figure S2.** Selection of highly invasive oral cancer cells, OC-3-IV and OC-3-IV-M, from OC-3 cells. **Figure S3.** Heat map of 36 most differentially regulated genes from cDNA microarrays of **(a)** OC-3 vs.OC-3-IV cells, **(b)** OC-3 vs.OC-3-IV-M cells and **(c)** OC-3-IV vs. OC-3-IV-M cells. **Figure S4.** Upregulation of KRT16 was found in highly invasive OSCC cells and was correlated with poor survival of the OSCC patients. **Figure S5.** Depletion of KRT16 leads to inhibition of migration, invasion and metastasis in OSCC cells and can be restored by ectopic expression of KRT16. **Figure S6.** Depletion of KRT16 leads to decreased cancer stemness. **Figure S7. (a)** The expression levels of EHF and KRT16 mRNAs in CGHNC9 and C9-IV3 lines were measured using qRT-PCR (**P < 0.01). **(b)** qRT-PCR of KRT16 mRNA in CGHNC9 cells transfected with the EHF-siRNAs or NC-siRNA. **Figure S8.** OC-3-IV- and OC-3-IV-M-shEHF-mediated inhibition of OSCC cell migration and invasion can be rescued by ectopic expression of KRT16. **Figure S9.** Four miRNAs were predicted to target potential EHF gene. **Figure S10.** The effects of miR-365-3p on EHF and KRT16-mediated migration and invasion in OC-3-IV- and OC-3-IV-M-pPG-GFP-miR-365-3p stable cells. **Figure S11.** KRT16 depletion enhances degradation of β5-integrin and c-Met in OSCC cells. **Figure S12.** MiR-365-3p/EHF/KRT16 signaling pathway could stimulate c-Met to transmit downstream signaling through β5-integrin. **Figure S13.** C-Met partially associates with KRT16 through β5-integrin and these three proteins may colocalize in OSCC cells. **Figure S14.** The mRNA expression levels of KRT16, β5-integrin (ITGB5) and c-Met correlate with overall survival in 56 OSCC patients as calculated from the clinical data from Chang Gung Memorial Hospital-Linkou in Taiwan. **Figure S15.** KRT16 depletion leads to autophagy activation to promote the endocytosis of c-Met. **Figure S16.** The effect of KRT16, c-Met and β5-integrin (ITGB5) on downstream Src/STAT3 signaling. **Figure S17.** Treatment with inhibitors of Src or JAK2 in KRT16 over-expressing OC-3-IV-M cells. **Figure S18.** 5-FU and genistein inhibited activation of c-Met/Src signaling in OC-3-IV cells. **Figure S19.** Inhibition of KRT16/β5-integrin/c-Met signaling enhances cytotoxicity of 5-FU treatment in OSCC cells. **Table S1.** Primers and siRNAs used in this study. **Table S2.** Oligonucleotide sequences used for qRT-PCR. (DOCX 12216 kb)

## Abbreviations

3’UTR: 3′-untranslated region
5-FU: 5-fluorouracil
CSC: Cancer stem-like cell
EHF: ETS homologous factor
HDs: Hemidesmosomes
HGF: Hepatocyte growth factor
ITGB5: β5-integrin
KRT16: Keratin 16 KRT16
LC3: Microtubule-associated protein 1A/1B-light chain 3
OSCC: Oral squamous cell carcinoma
RTKs: Receptor tyrosine kinases
