# Supplementary material for: A novel miR-365-3p/EHF/keratin 16 axis promotes oral squamous cell carcinoma metastasis, cancer stemness and drug resistance via enhancing β5-integrin/c-met signaling pathway
Source: J Exp Clin Cancer Res. 2019 Feb 19;38:89. doi: 10.1186/s13046-019-1091-5 (PMC6381632; doi:10.1186/s13046-019-1091-5)
Supplement: Supplementary file 1 — Figure S1. Establishment of shKRT16, shEHF and miR-365-3p stably expressing cancer cell lines in invasive OSCC lines. Figure S2. Selection of highly invasive oral cancer cells, OC-3-IV and OC-3-IV-M, from OC-3 cells. Figure S3. Heat map of 36 most differentially regulated genes from cDNA microarrays of (a) OC-3 vs.OC-3-IV cells, (b) OC-3 vs.OC-3-IV-M cells and (c) OC-3-IV vs. OC-3-IV-M cells. Figure S4. Upregulation of KRT16 was found in highly invasive OSCC cells and was correlated with poor survival of the OSCC patients. Figure S5. Depletion of KRT16 leads to inhibition of migration, invasion and metastasis in OSCC cells and can be restored by ectopic expression of KRT16. Figure S6. Depletion of KRT16 leads to decreased cancer stemness. Figure S7. (a) The expression levels of EHF and KRT16 mRNAs in CGHNC9 and C9-IV3 lines were measured using qRT-PCR (**P < 0.01). (b) qRT-PCR of KRT16 mRNA in CGHNC9 cells transfected with the EHF-siRNAs or NC-siRNA. Figure S8. OC-3-IV- and OC-3-IV-M-shEHF-mediated inhibition of OSCC cell migration and invasion can be rescued by ectopic expression of KRT16. Figure S9. Four miRNAs were predicted to target potential EHF gene. Figure S10. The effects of miR-365-3p on EHF and KRT16-mediated migration and invasion in OC-3-IV- and OC-3-IV-M-pPG-GFP-miR-365-3p stable cells. Figure S11. KRT16 depletion enhances degradation of β5-integrin and c-Met in OSCC cells. Figure S12. MiR-365-3p/EHF/KRT16 signaling pathway could stimulate c-Met to transmit downstream signaling through β5-integrin. Figure S13. C-Met partially associates with KRT16 through β5-integrin and these three proteins may colocalize in OSCC cells. Figure S14. The mRNA expression levels of KRT16, β5-integrin (ITGB5) and c-Met correlate with overall survival in 56 OSCC patients as calculated from the clinical data from Chang Gung Memorial Hospital-Linkou in Taiwan. Figure S15. KRT16 depletion leads to autophagy activation to promote the endocytosis of c-Met. Figure S16. The effect [file 13046_2019_1091_MOESM1_ESM.docx]

**Additional file 1**

**A novel miR-365-3p/EHF/Keratin16 axis promotes oral squamous cell carcinoma metastasis, cancer stemness and drug resistance via enhancing β5-integrin/c-Met signaling pathway**

Wei-Chieh Huang, Te-Hsuan Jang, Shiao-Lin Tung, Tzu-Chen Yen, Shih-Hsuan Chan and Lu-Hai Wang

**Additional file 1: Figures**


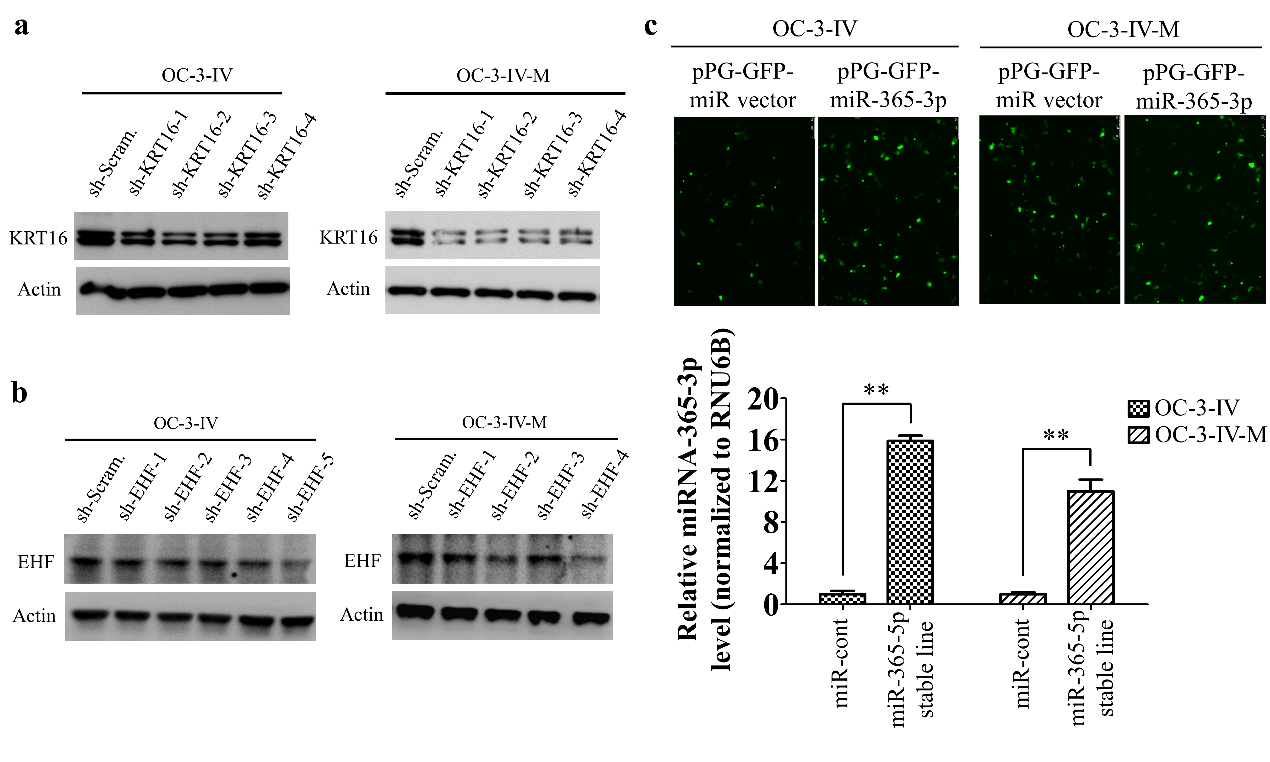


### Figure S1. Establishment of shKRT16, shEHF and miR-365-3p stably expressing cancer cell lines in invasive OSCC lines.

**(a)** Immunoblotting of KRT16 protein level in OC-3-IV- and OC-3-IV-M-shKRT16 stable lines. **(b)** Immunoblotting of EHF protein level in OC-3-IV- and OC-3-IV-M-shEHF stable lines. **(c)** Immunofluorescence (top) and qRT-PCR (bottom) showed that miR-365-3p level was up-regulated in OC-3-IV- and OC-3-IV-M-pPG-GFP-miR-365-3p stable lines.


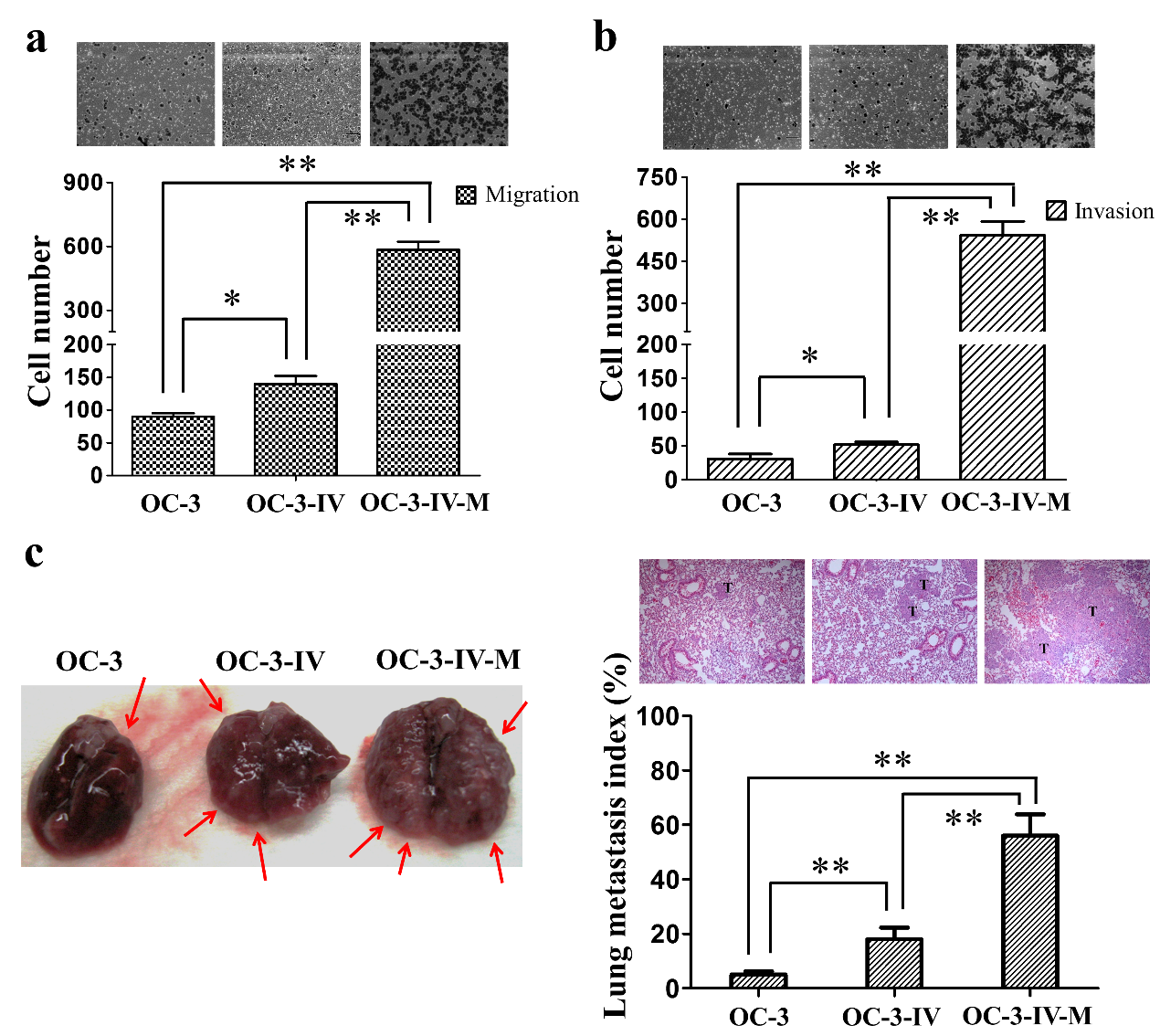


Figure **S2.** Selection of highly invasive oral cancer cells, OC-3-IV and OC-3-IV-M, from OC-3 cells.

The **(a)** migration and **(b)** invasion ability of the *in vivo* selected highly invasive cells was compared with that of their parental cells (OC-3 cells). The first round of *in vivo* selection of lung metastasis of OC-3 was done using tail vein injection. 5 x 10^5^ OC-3 cells were injected into CB17-SCID mice through tail veins; after growing metastatic lung tumors for 12 months, these tumors were isolated to obtain the OC-3-IV subline. The OC-3-IV cells were reinjected into the tail veins of CB17-SCID mice; after growing metastatic lung tumors for 2 months, these tumors were isolated to obtain the OC-3-IV-M subline. **(c)** Lung metastasis via tail vein injection of OC-3-IV-M cells into CB17-SCID mice was significantly increased compared with OC-3 and OC-3-IV cells. The original magnification was 40x. Histograms represent means ± SD from three independent experiments (*, *P* < 0.05, **, *P* < 0.01).

**
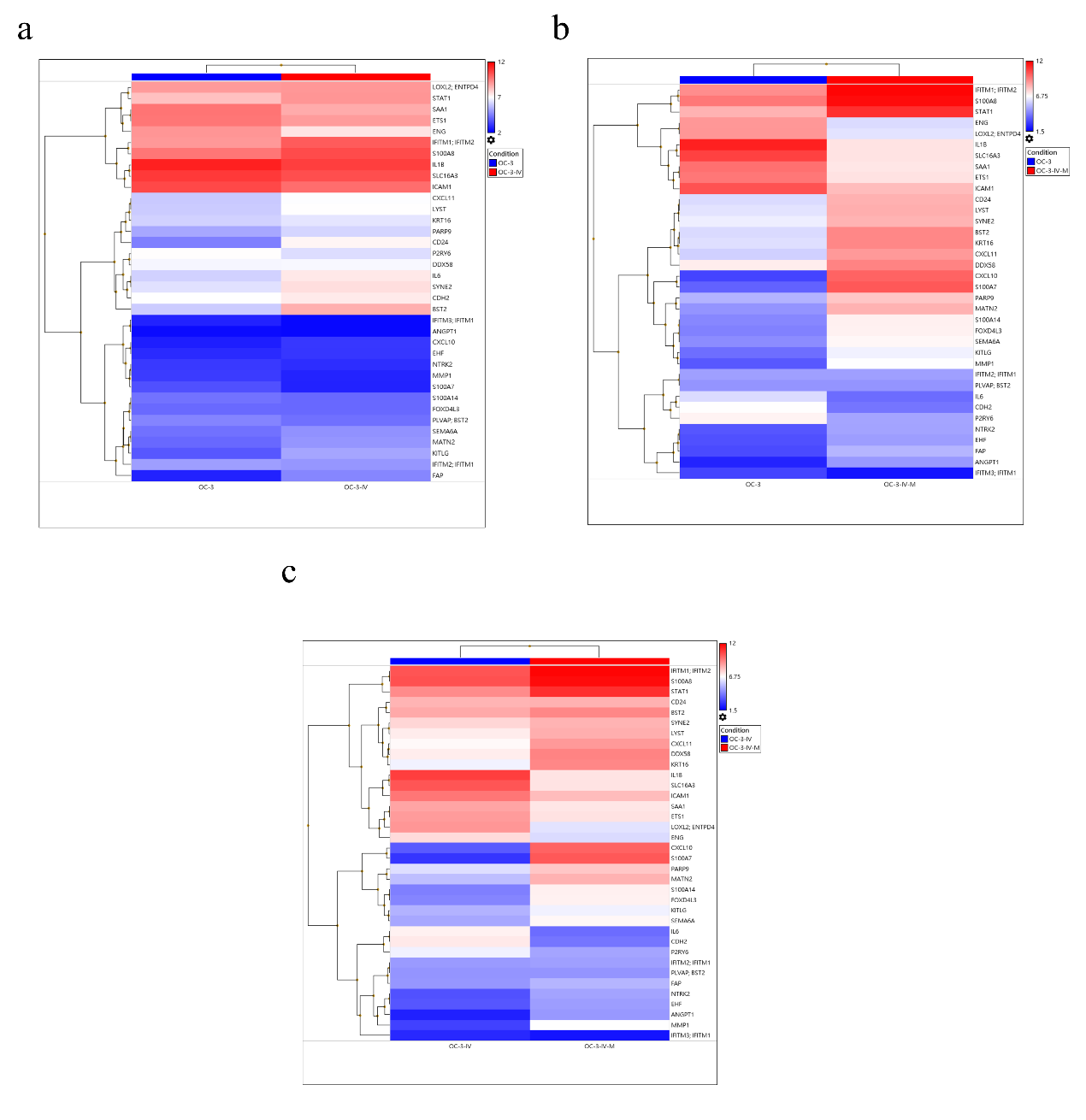
**

Figure **S3.** Heat map of 36 most differentially regulated genes from cDNA microarrays of **(a)** OC-3 vs.OC-3-IV cells, **(b)** OC-3 vs.OC-3-IV-M cells and **(c)** OC-3-IV vs. OC-3-IV-M cells.


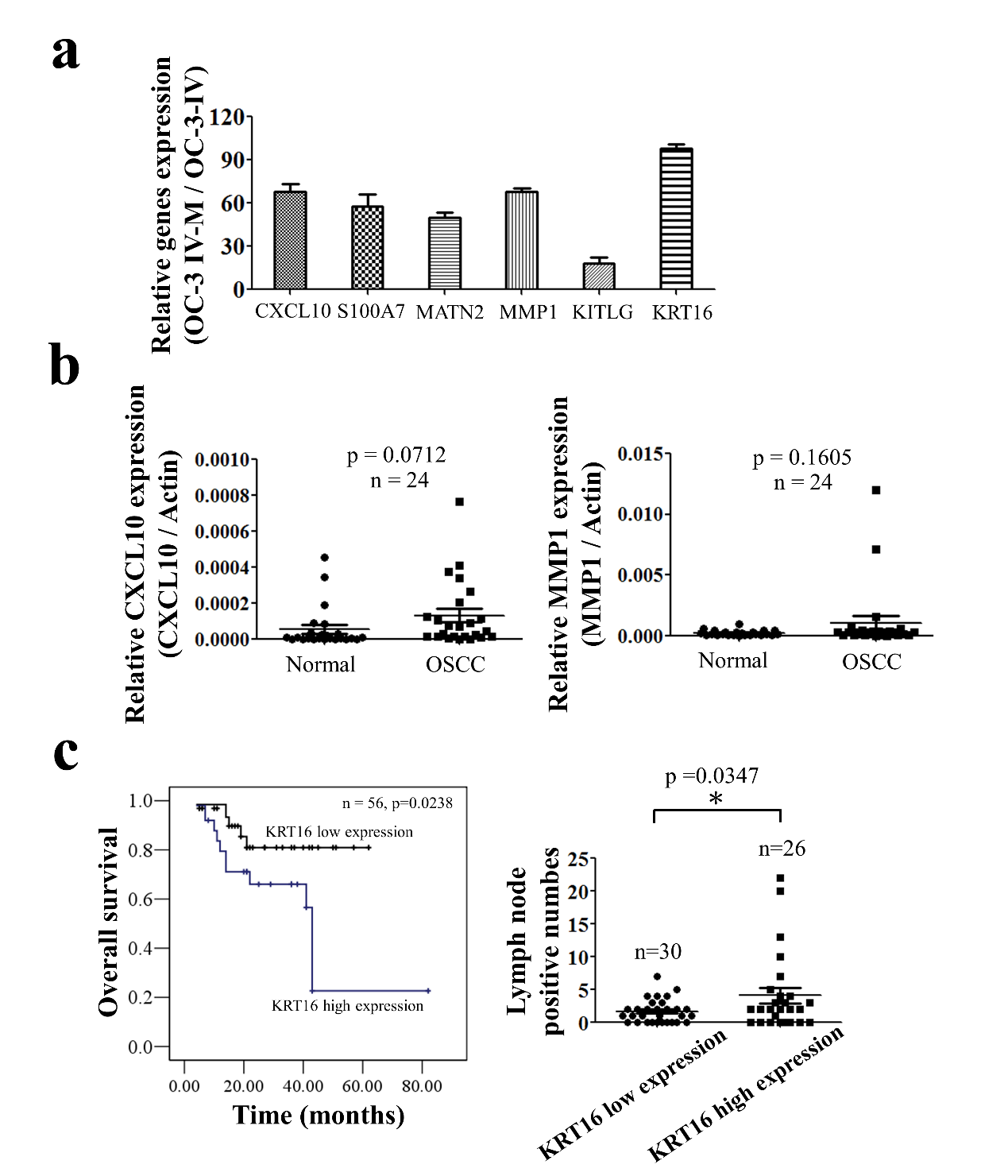


Figure **S4.** Upregulation of KRT16 was found in highly invasive OSCC cells and was correlated with poor survival of the OSCC patients.

**(a)** The relative mRNA expression ratios of top six genes in OC-3-IV-M cell when compared with OC-3-IV cells were analyzed by qRT–PCR with actin as an internal control. **(b)** qRT–PCR of mRNAs of CXCL10 (p = 0.0712) and MMP1 (p = 0.1605) in OSCC tissues compared with their matched normal oral tissues. **(c)** Left, Kaplan-Meier survival analysis based on cDNA microarray data from 56 OSCC tumor samples from Chang Gung Memorial Hospital-Linkou in Taiwan. Patients were divided into high and low expression groups based on the median expression of the KRT16. Right, the cohort data of the 56 sample patients were stratified according to the number of lymph node metastasis. The data revealed a positive correlation between increased number of lymph nodes metastasis and high KRT16 expression. The t-test analysis was used to evaluate the relationship of KRT16 expression and lymph node metastasis (*, *P* < 0.05).

**
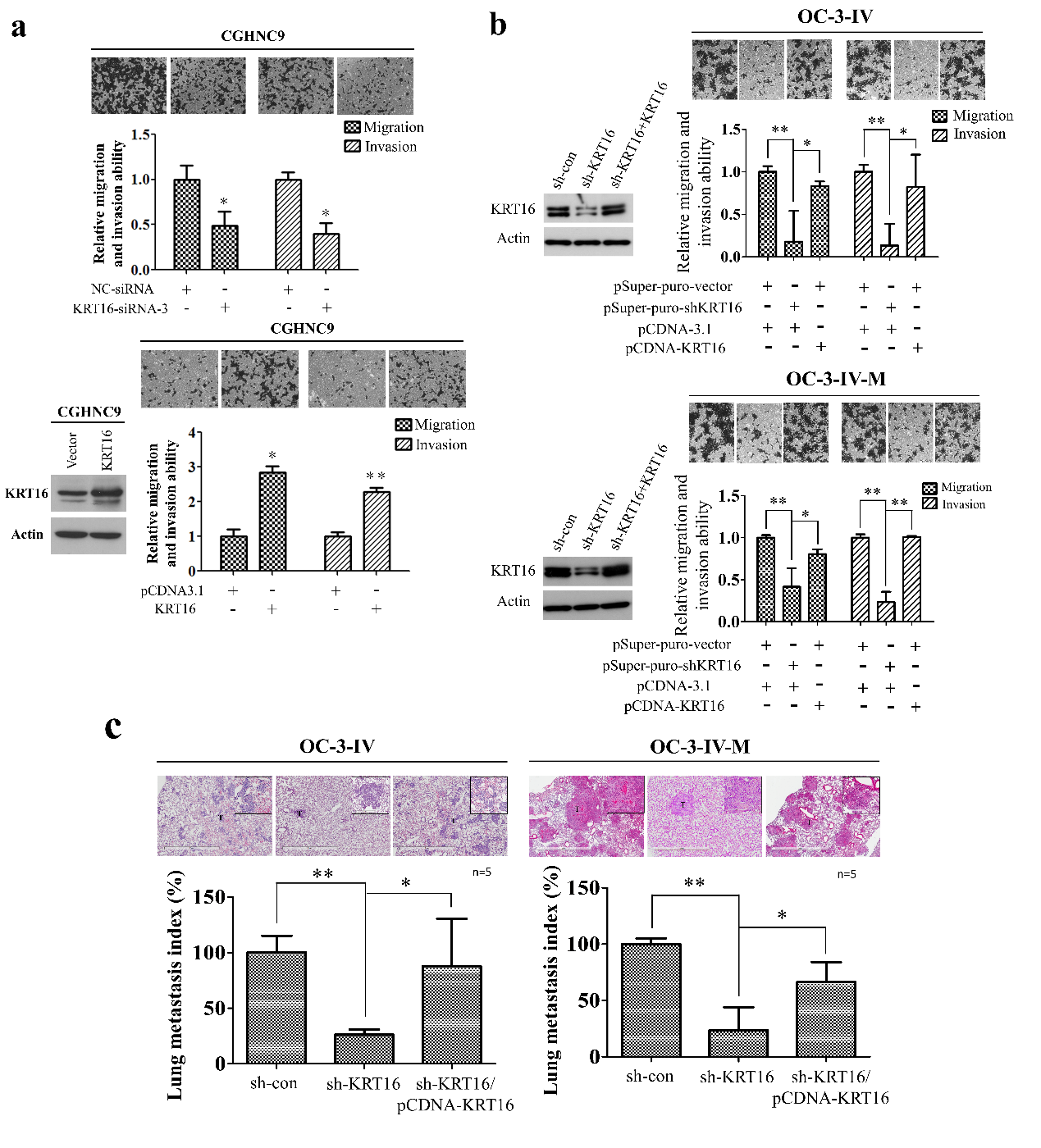
**

Figure **S5.** Depletion of KRT16 leads to inhibition of migration, invasion and metastasis in OSCC cells and can be restored by ectopic expression of KRT16.

**(a)** Top, a decrease in migration and invasion abilities was observed in CGHNC9 cells transfected with the KRT16-siRNA-3 compared with the control (NC-siRNA). Bottom, left, Western blotting of KRT16 from CGHNC9 cells transfected with the KRT16 plasmid or control vector. Bottom, right, increased cell migration and invasion abilities were observed by ectopic expression of KRT16 in CGHNC9 cells. **(b)** Left, immunoblotting of KRT16 from OC-3-IV and OC-3-IV-M shKRT16 stable cells transfected with the KRT16 plasmid or control vector. Right, cell migration and invasion were restored through ectopic expression of KRT16 in OC-3-IV- and OC-3-IV-M-shKRT16 stable cells. **(c)** The lung metastatic ability decreased in stable shKRT16 line whereas ectopic expression of KRT16 partially reversed the KRT16 depletion-mediated inhibition.


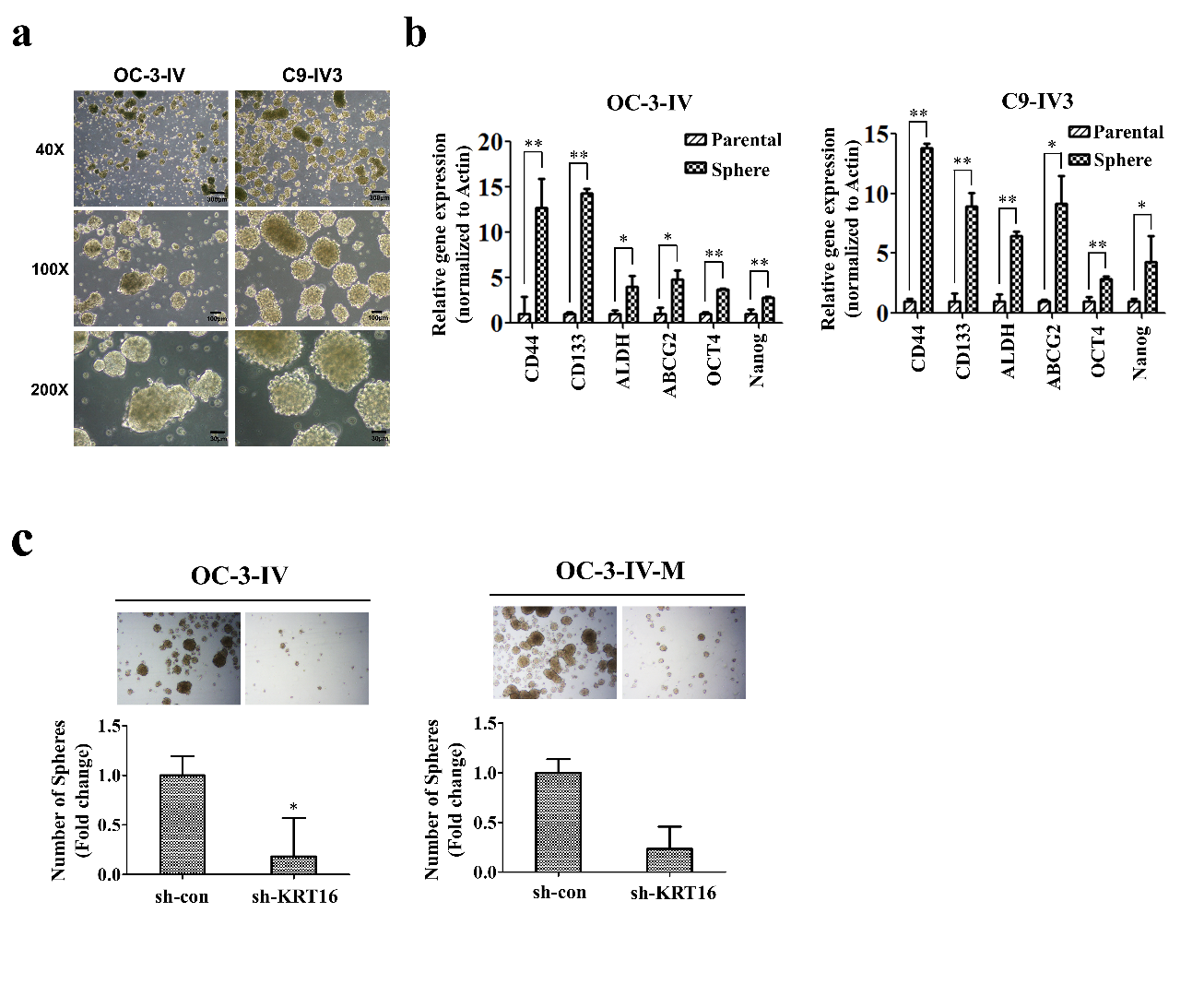


Figure **S6.** Depletion of KRT16 leads to decreased cancer stemness.

**(a)** Oral CSCs were enriched from OC-3-IV and C9-IV3 cell lines. Formation of spheres under the stem cell selective condition on day 8 after culturing from parental OC-3-IV and C9-IV3 lines is shown. The spheres grew larger and assumed a confluent rounded three-dimensional configuration after 4–8 days cultured in the stem cell selective condition. The original magnification was 40x, 100x, and 200x, respectively. **(b)** Sphere cells enriched from parental OC-3-IV and C9-IV3 lines expressed important CSC markers shown by qRT-PCR. Histograms represent means ± SD from three independent experiments (*, *P* < 0.05, **, *P* < 0.01). **(c)** Significant reduction of sphere formation was observed in C-3-IV- and OC-3-IV-M-shKRT16 stable cells.


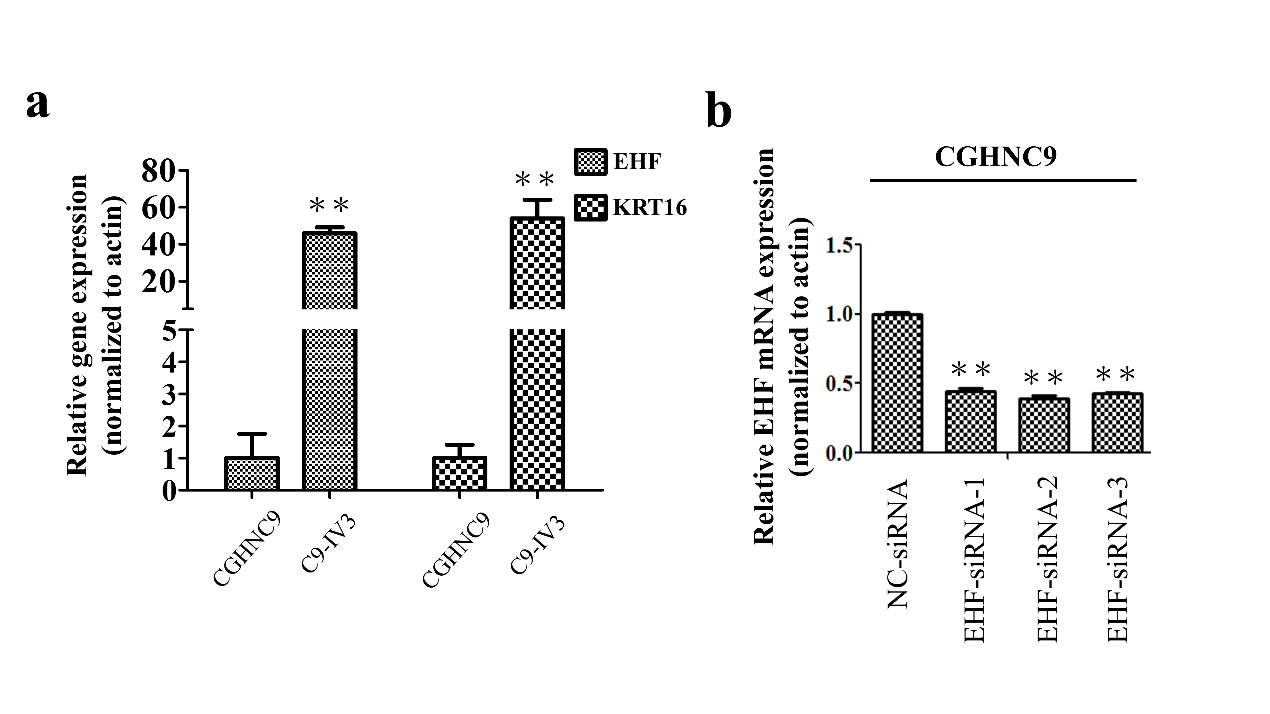


Figure **S7. (a)** The expression levels of EHF and KRT16 mRNAs in CGHNC9 and C9-IV3 lines were measured using qRT-PCR (**P< 0.01). **(b)** qRT-PCR of KRT16 mRNA in CGHNC9 cells transfected with the EHF-siRNAs or NC-siRNA.

**
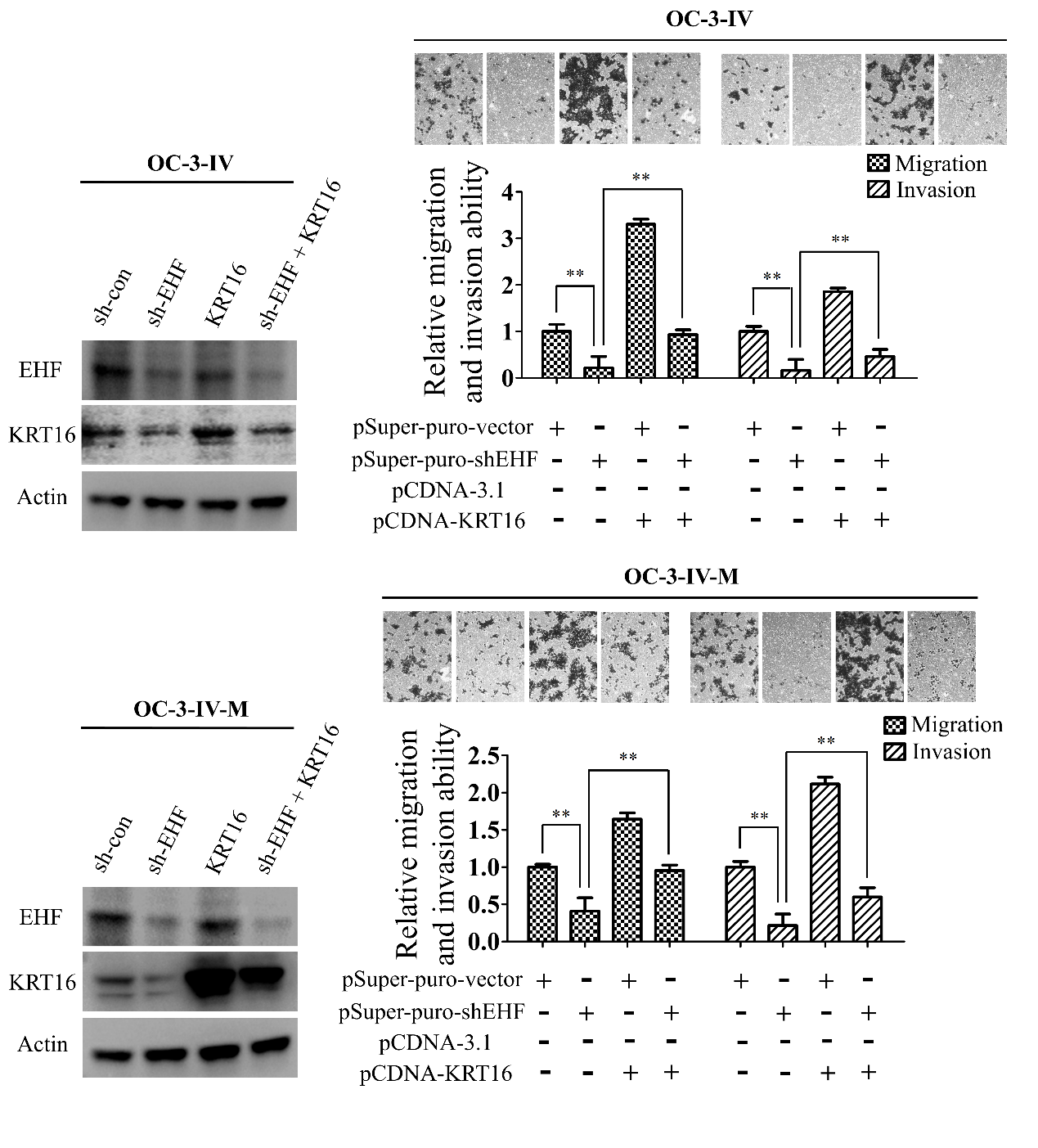
** Figure **S8.** OC-3-IV- and OC-3-IV-M-shEHF-mediated inhibition of OSCC cell migration and invasion can be rescued by ectopic expression of KRT16.

OC-3-IV and OC-3-IV-M cells were transfected with pSuper-puro or pSuper-puro-shEHF and clonal selection was carried out by screening for puromycin (Sigma-Aldrich) resistance to obtain stable expressing cancer cell lines. Next, the stably shEHF cell lines were transfected with pCDNA-KRT16 by liposome transfection, we performed G418 (Sigma-Aldrich)/puromycin (Sigma-Aldrich) resistance screening for approximately two weeks to obtain stable expressing cancer cell lines. Left, immunoblotting showed that the expression of KRT16 protein was increased in OC-3-IV- and OC-3-IV-M-shEHF stable cells by ectopic expression of KRT16. Right, the migration and invasion abilities were decreased in shEHF cells and were partially restored partially by ectopic expression of KRT16.


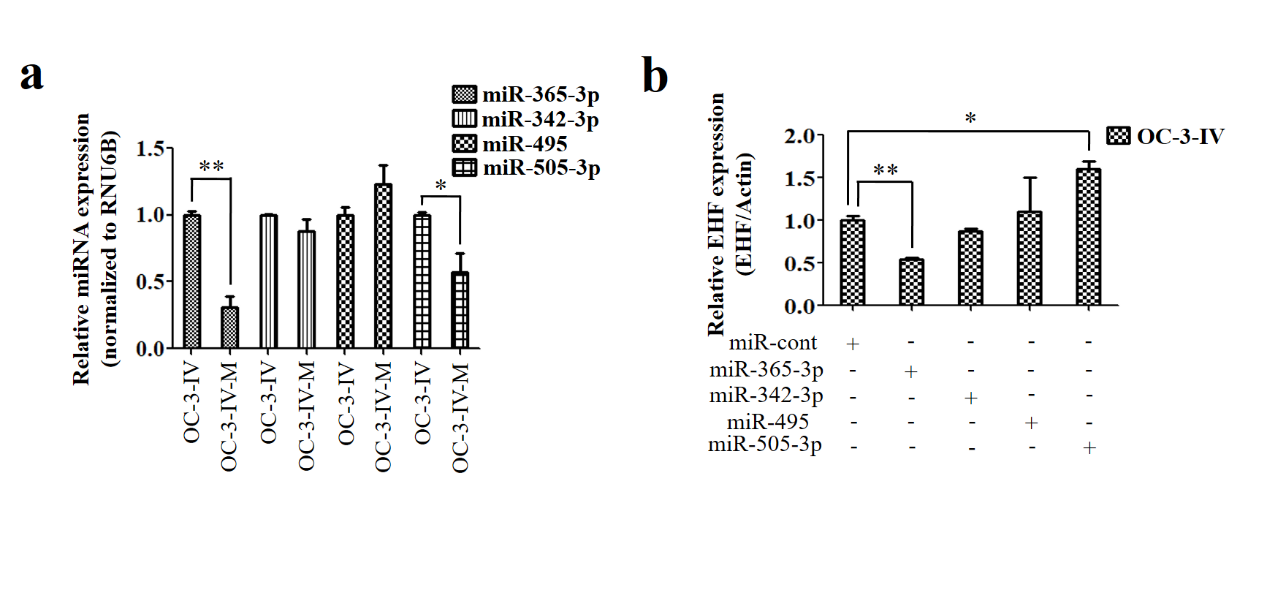


Figure **S9.** Four miRNAs were predicted to target potential EHF gene.

**(a)** The qRT-PCR of four miRNAs predicted to target potential EHF gene in OC-3-IV-M cells compared with OC-3-IV cell. **(b)** miR-365-3p inhibited EHF mRNA expression levels in OC-3-IV cells. Histograms represent means ± SD from three independent experiments (*, *P* < 0.05, **, *P* < 0.01).

**
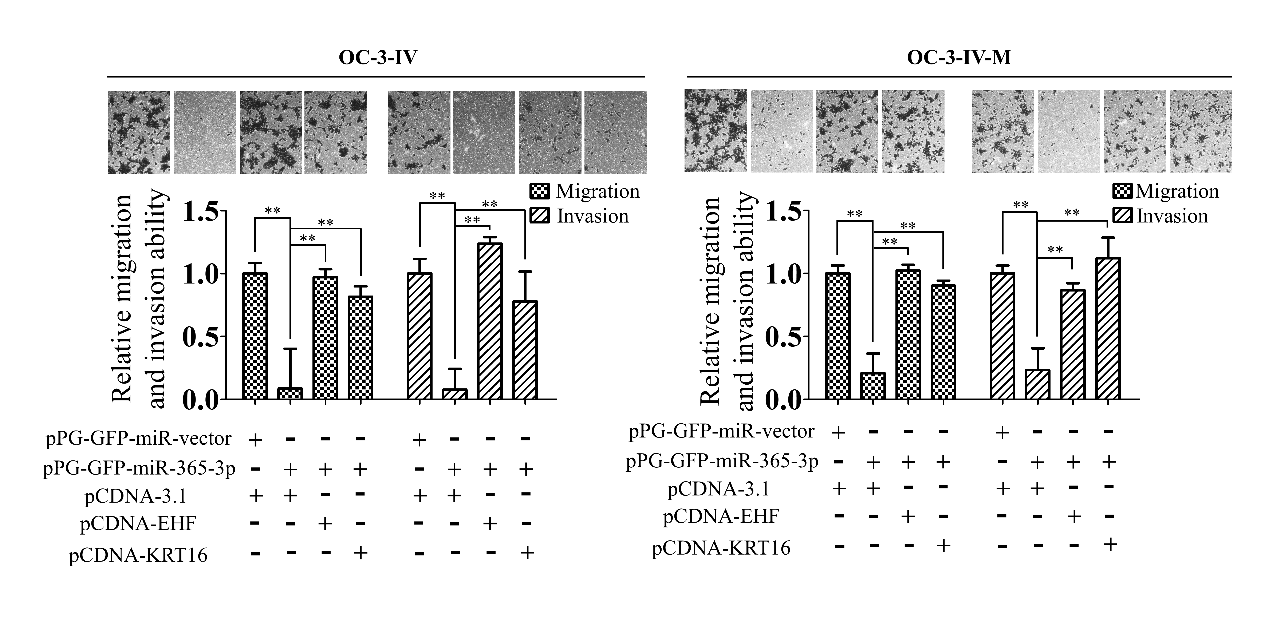
**Figure **S10.** The effects of miR-365-3p on EHF and KRT16-mediated migration and invasion in OC-3-IV- and OC-3-IV-M-pPG-GFP-miR-365-3p stable cells.


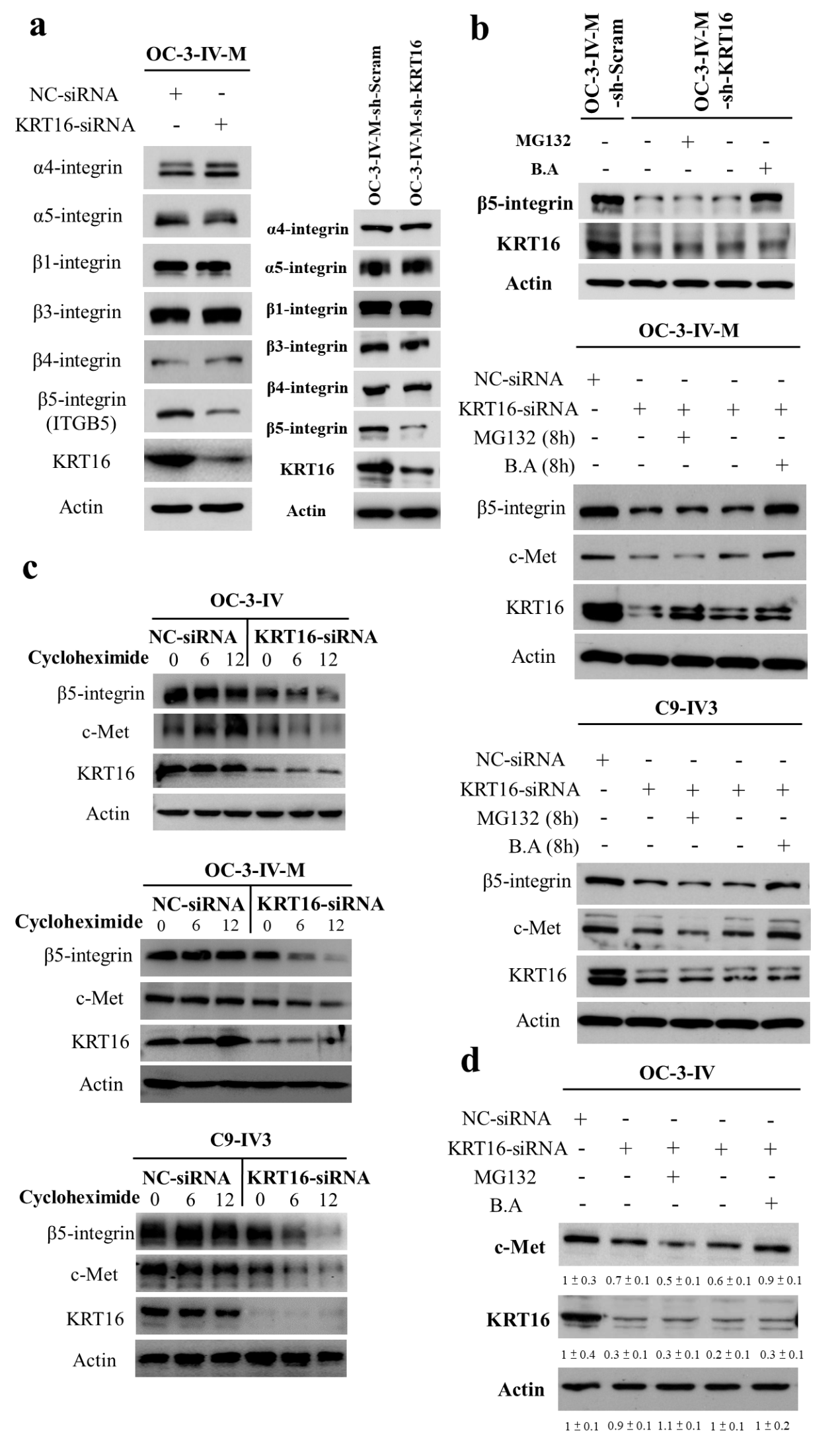


Figure **S11.** KRT16 depletion enhances degradation of β5-integrin and c-Met in OSCC cells.

**(a)** Analysis of integrin isoforms in KRT16-siRNA–transfected OC-3-IV-M cells and OC-3-IV-M-shKRT16 stable lines. (**b)** Treatment with 10 μM MG132 for 8 h could not prevent the decrease of β5-integrin (ITGB5) and c-Met in KRT16-depleted cells. Treatment with 100 nM bafilomycin A (B.A.) for 8 h prevented β5-integrin and c-Met degradation in KRT16-depleted OSCC cells. Knockdown of KRT16 enhanced degradation of c-Met in OC-3-IV-M and C9-IV3 cells. **(c)** The β5-integrin and c-Met levels in control and KRT16-depleted OC-3-IV, OC-3-IV-M and C9-IV3 cells treated with cycloheximide (20 μg/mL) for the indicated times showed a faster decay in the KRT16 depleted cells. **(d)** Treatment with 10 μM MG132 for 8 h could not prevent the loss of c-Met in KRT16-depleted cells. Treatment with 100 nM bafilomycin A (B.A.) for 24 h prevented c-Met degradation in KRT16-depleted cells.


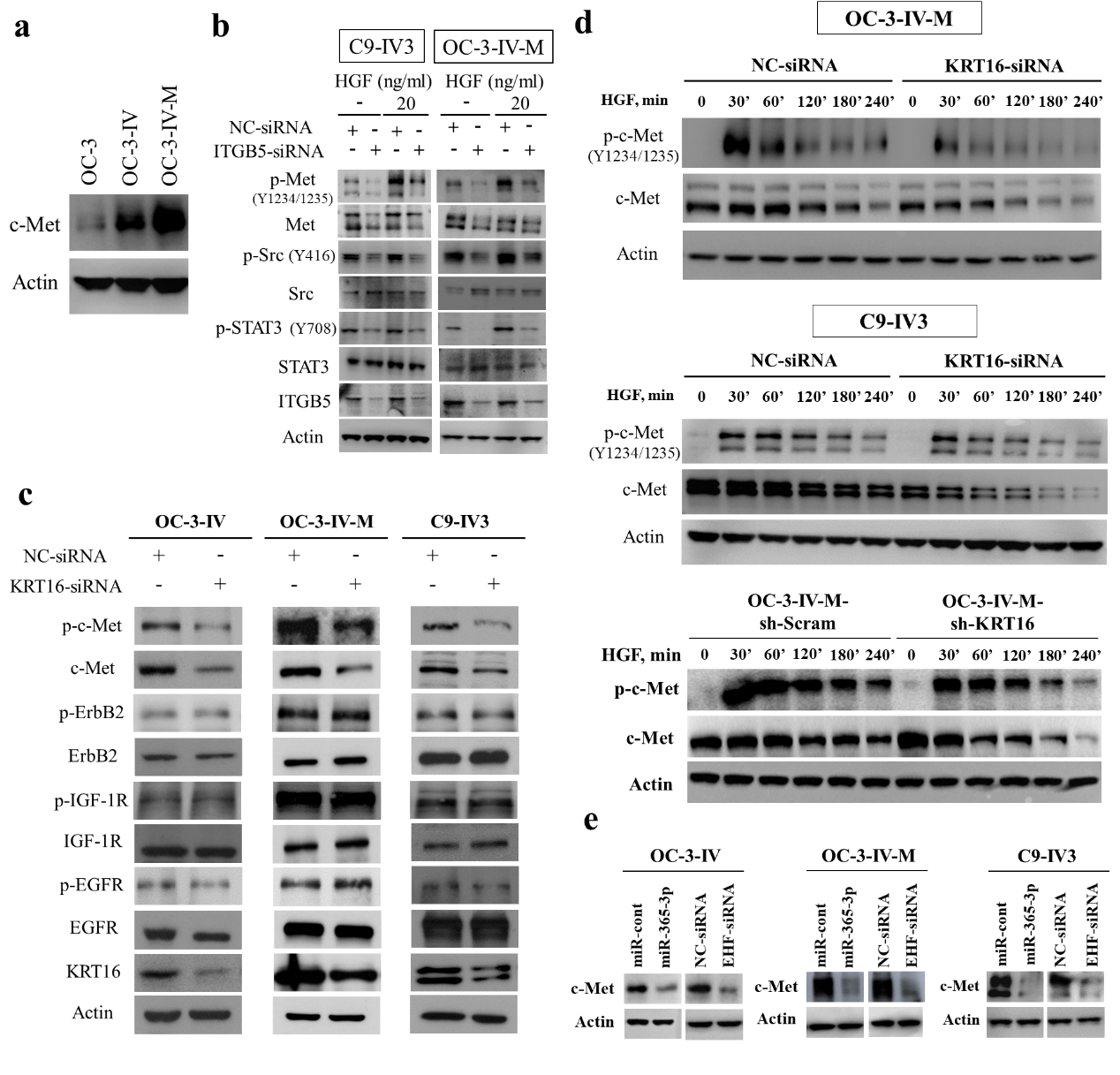


Figure **S12.** miR-365-3p/EHF/KRT16 signaling pathway could stimulate c-Met to transmit downstream signaling through β5-integrin.

**(a)** Immunoblotting showed increased c-Met protein expression levels in highly invasive lines, OC-3-IV and OC-3-IV-M, compared with their parental OC-3 line. **(b)** NC-siRNA and β5-integrin-silenced C9-IV3 and OC-3-IV-M cells were serum starved for 18h, then treated with 20 ng/mL HGF for 30 min, and subjected to immunoblotting. **(c)** Immunoblotting showed the proteins and their phosphorylated proteins expression level of different RTKs in KRT16-siRNA-transfected OC-3-IV, OC-3-IV-M and C9-IV3 cells. **(d)** OC-3-IV-M stably expressing shKRT16 or empty vector, OC-3-IV, OC-3-IV-M and C9-IV3 cells were transfected with the indicated plasmids for 8 h, serum starved for 24 h, and then were treated with 50 ng/mL HGF for the indicated times. **(e)** Immunoblotting showed c-Met expression levels in miR-365-3p transfected- and EHF-siRNA-transiently transfected OSCC cells.

**
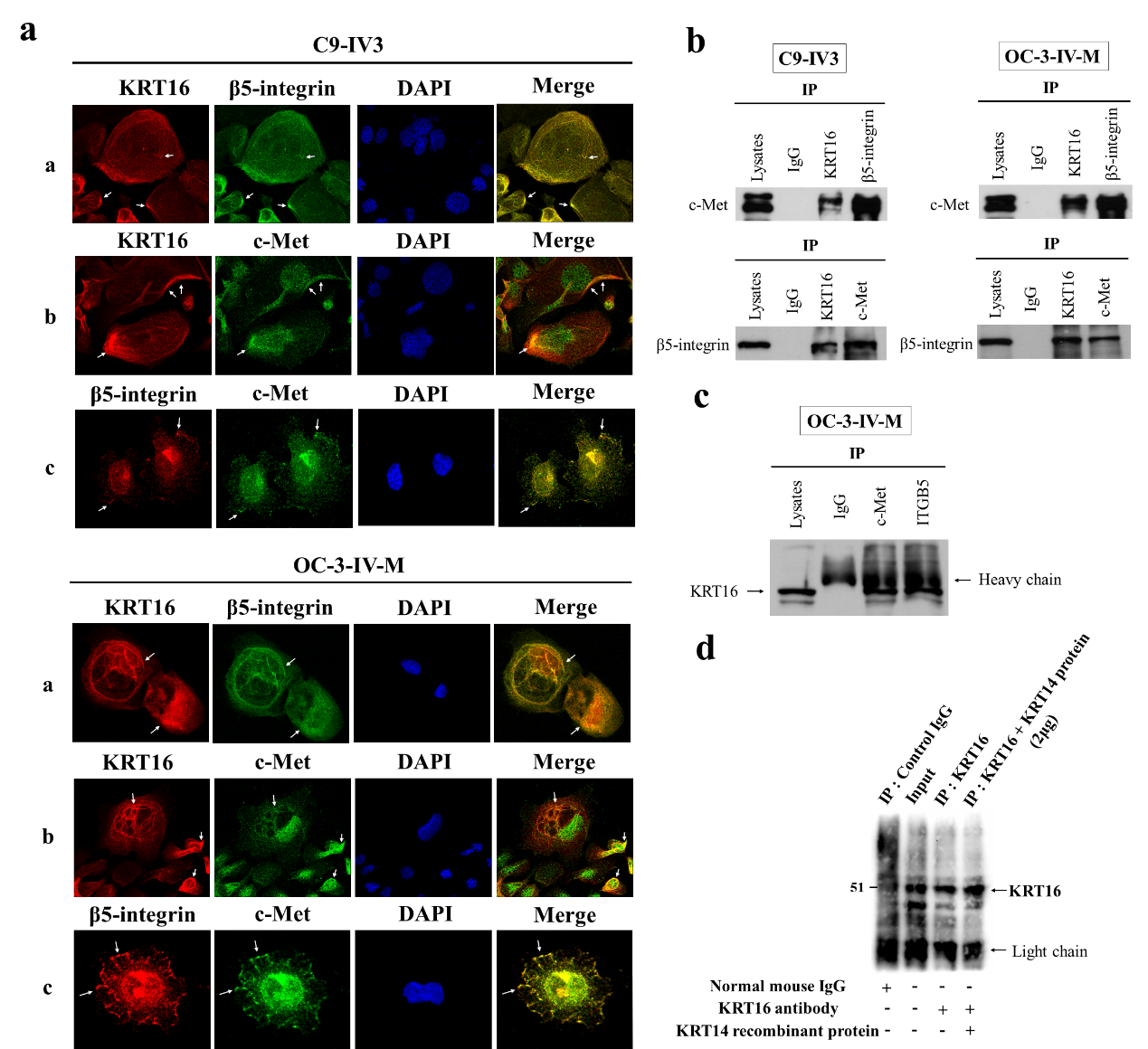
**

Figure **S13.** c-Met partially associates with KRT16 through β5-integrin and these three proteins may colocalize in OSCC cells.

**(a)** Localization of KRT16, c-Met, and β5-integrin was analyzed in C9-IV3 and OC-3-IV-M cells through confocal microscopy. Panel a, IF staining under confocal microscopy of KRT16 (red), β5-integrin (ITGB5) (green), and nuclei (DAPI, blue) after adhesion of OSCC cells to Poly-L-Lysine; panel b, IF staining under confocal microscopy of KRT16 (red), c-Met (green), and nuclei (DAPI, blue); panel c, IF staining under confocal microscopy of ITGB5 (red), c-Met (green), and nuclei (DAPI, blue). (b) and (c) C9-IV3 and OC-3-IV-M cell lysates were blotted directly or subjected to IP with the indicated antibodies followed by blotting with the indicated antibodies. IgG served as the negative control. **(d)** OC-3-IV-M cell lysates were blotted directly or subjected to IP with the KRT16 antibody with or without KRT14 recombinant protein (2μg) (Protein Specialists, Inc.) added to cell lysates for possible competition of binding to antibody, followed by western blotting with the KRT16 antibody. Co-IP was performed using KRT16 antibody (SC-53255, SANTA CRUZ) and immunoblotted with rabbit-KRT16 antibody conjugated with HRP (ab76416, Abcam) and then with rabbit kappa-light chain HRP conjugated secondary antibody. IgG served as the negative control.

**
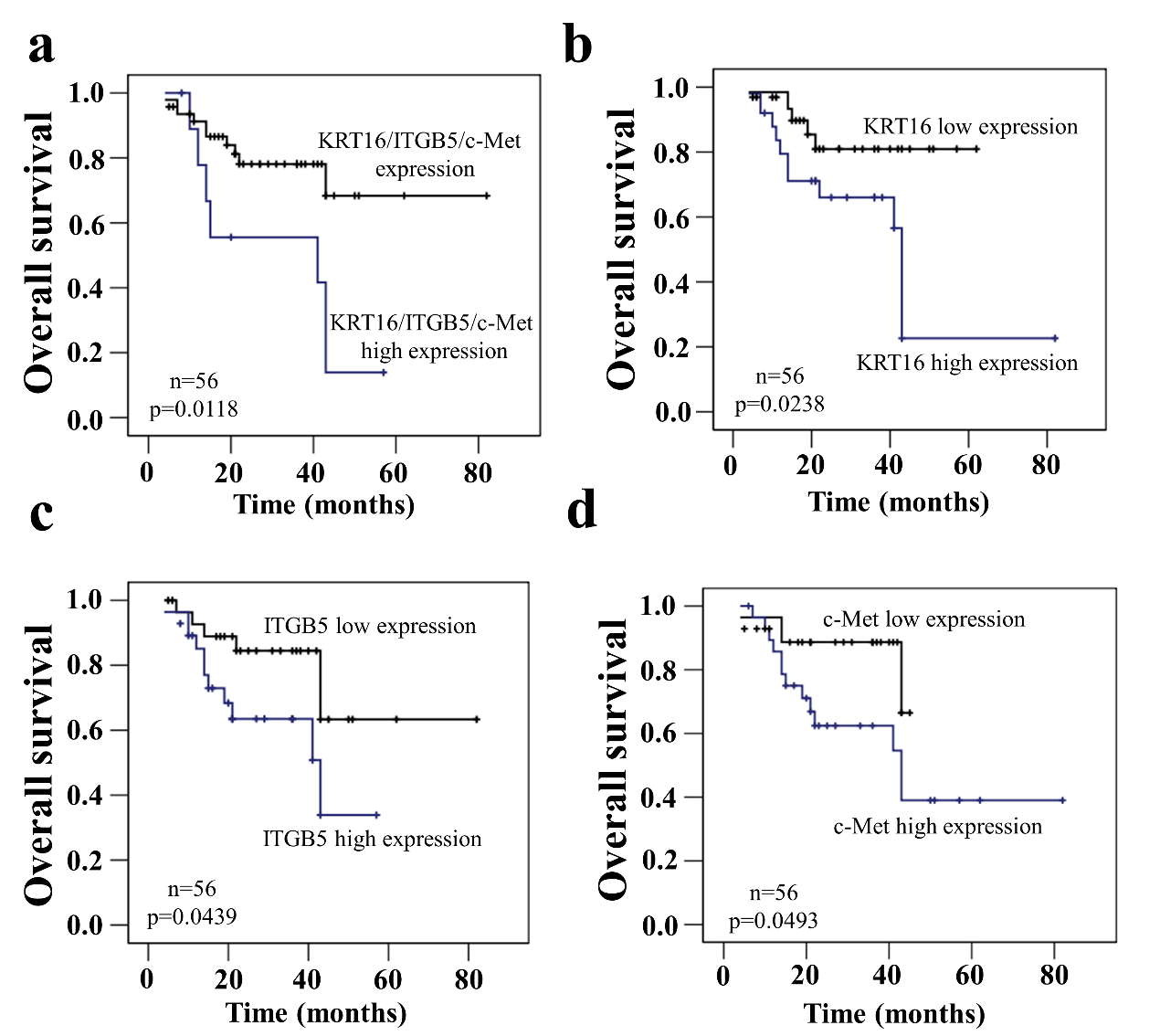
** Figure **S14.** The mRNA expression levels of KRT16, β5-integrin (ITGB5) and c-Met correlate with overall survival in 56 OSCC patients as calculated from the clinical data from Chang Gung Memorial Hospital-Linkou in Taiwan. **(a)** Higher expression level of these three genes (KRT16, ITGB5, c-Met) correlates with worse overall survival in OSCC patients. **(b) (c) (d)** Similar results were found in individual gene expression as indicated.


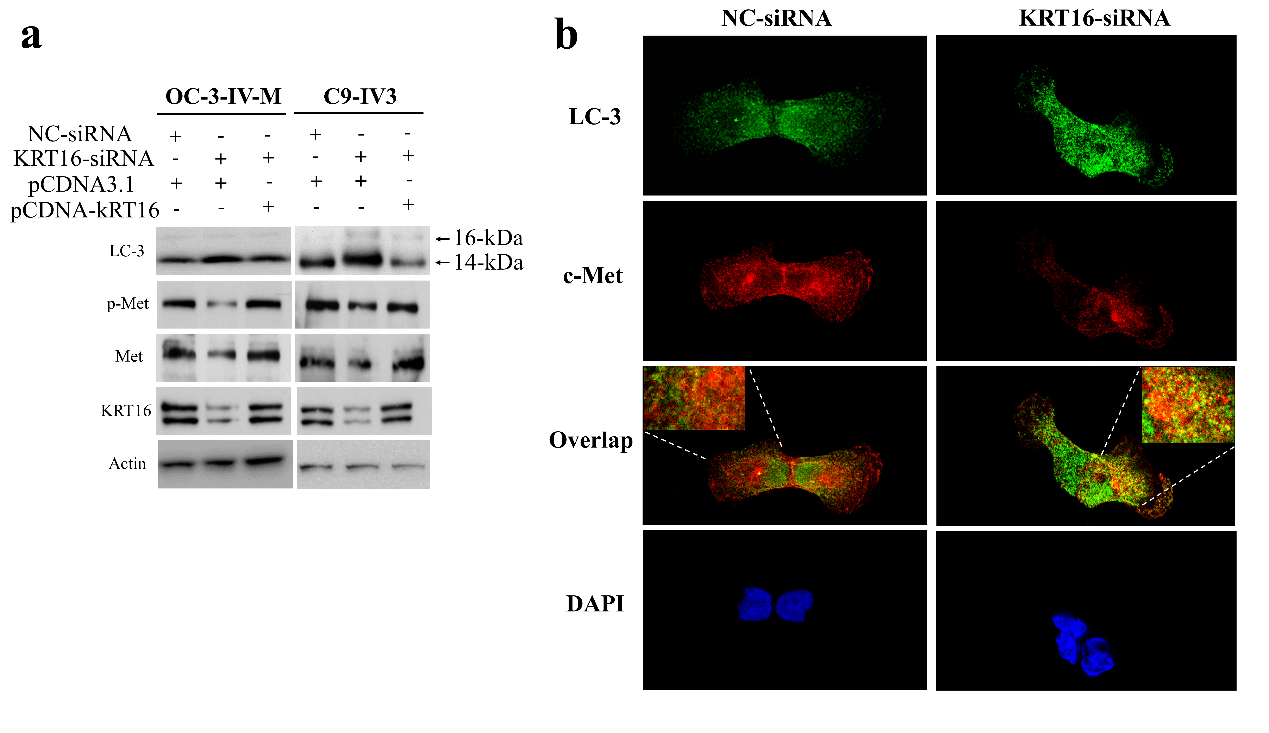


Figure **S15.** KRT16 depletion leads to autophagy activation to promote the endocytosis of c-Met

**(a)** Knockdown of KRT16 increased LC3 protein level and reduced phosphorylation of c-Met. **(b)** Immunofluorescence staining showed that the internalized c-Met (indicated by red fluorescence) were more co-localized with LC-3 (indicated by green fluorescence) in the KRT16 depleted OC3-IV-M cells compared with control cells.


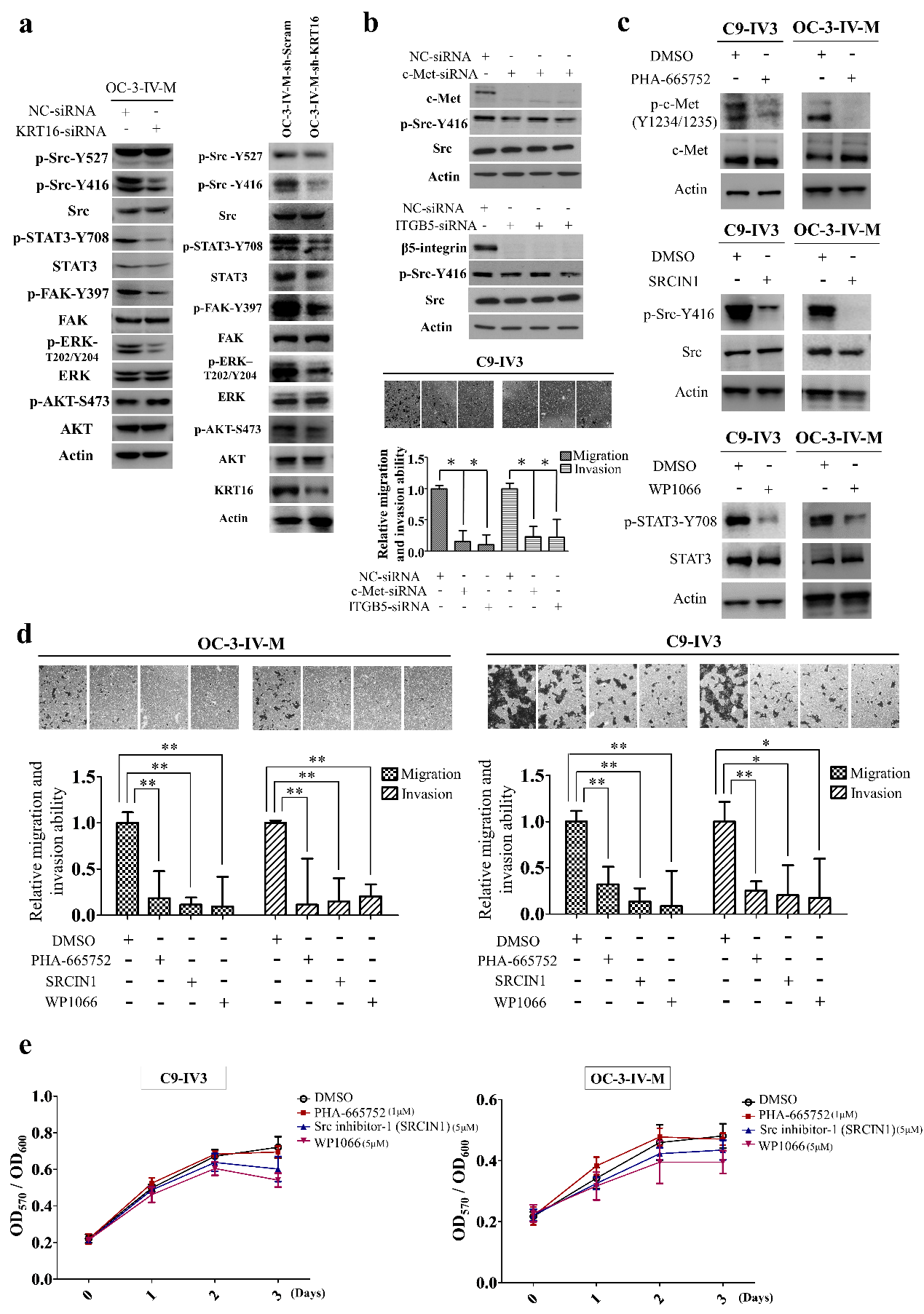


Figure **S16.** The effect of KRT16, c-Met and β5-integrin (ITGB5) on downstream Src/STAT3 signaling.

**(a)** Effect of KRT16 knockdown on c-Met signaling–related molecules in OC-3-IV-M cells. The actin was used as an internal control. **(b)** Top, Western blotting of c-Met, β5-integrin and p-Src-416 in C9-IV3 cells transfected with the indicated siRNA or negative control (NC-siRNA). Bottom, a dramatic decrease in migration and invasion ability was observed in C9-IV3 cells transfected with c-Met- or β5-integrin siRNA compared with the control. Histograms represent means ± SD from three independent experiments (*, *P* < 0.05, **, *P* < 0.01). **(c)** Immunoblotting showed levels of phosphorylation of c-Met, Src and STAT3 in C9-IV3 and OC-3-IV-M cells 24 h after treatments with c-Met inhibitor (30 nM), Src inhibitor (20 nM) and STAT3 inhibitor (50 nM). **(d)** OC-3-IV-M and C9-IV3 cells treated with inhibitor of c-Met, Src or STAT3 or with DMSO as a control were subjected to migration and invasion assays. **(e)** C9-IV3 and OC-3-IV-M cells were treated with PHA-665752 (c-Met inhibitor), SRCIN1 (Src inhibitor) and WP1066 (STAT3 inhibitor) or their negative control (DMSO), cell growth rates were detected by alamarBlue cell viability assay (Thermo Fisher Scientific), which is a colorimetric method for determining the viable cells in proliferation.


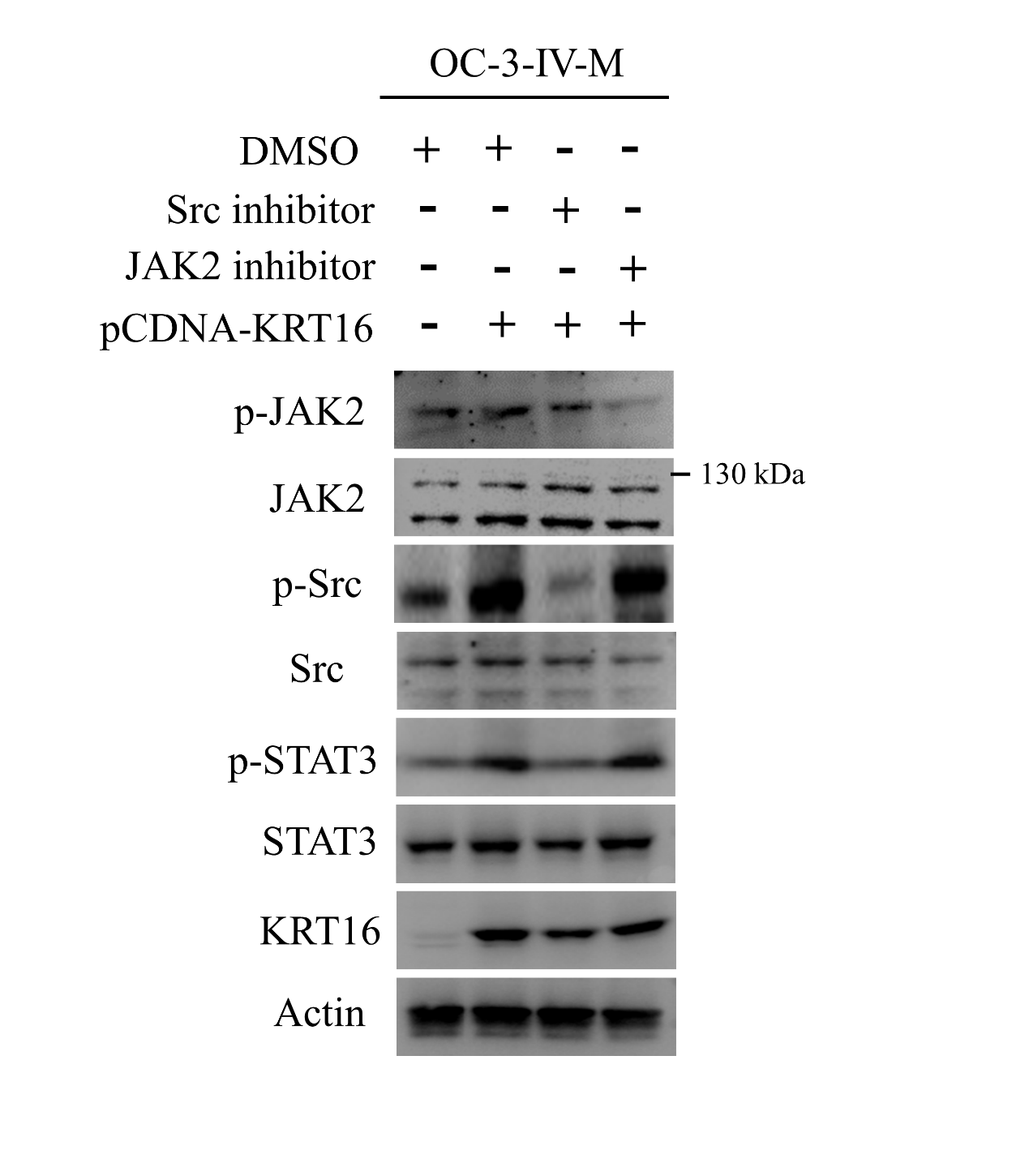


Figure **S17.** Treatment with inhibitors of Src or JAK2 in KRT16 over-expressing OC-3-IV-M cells.


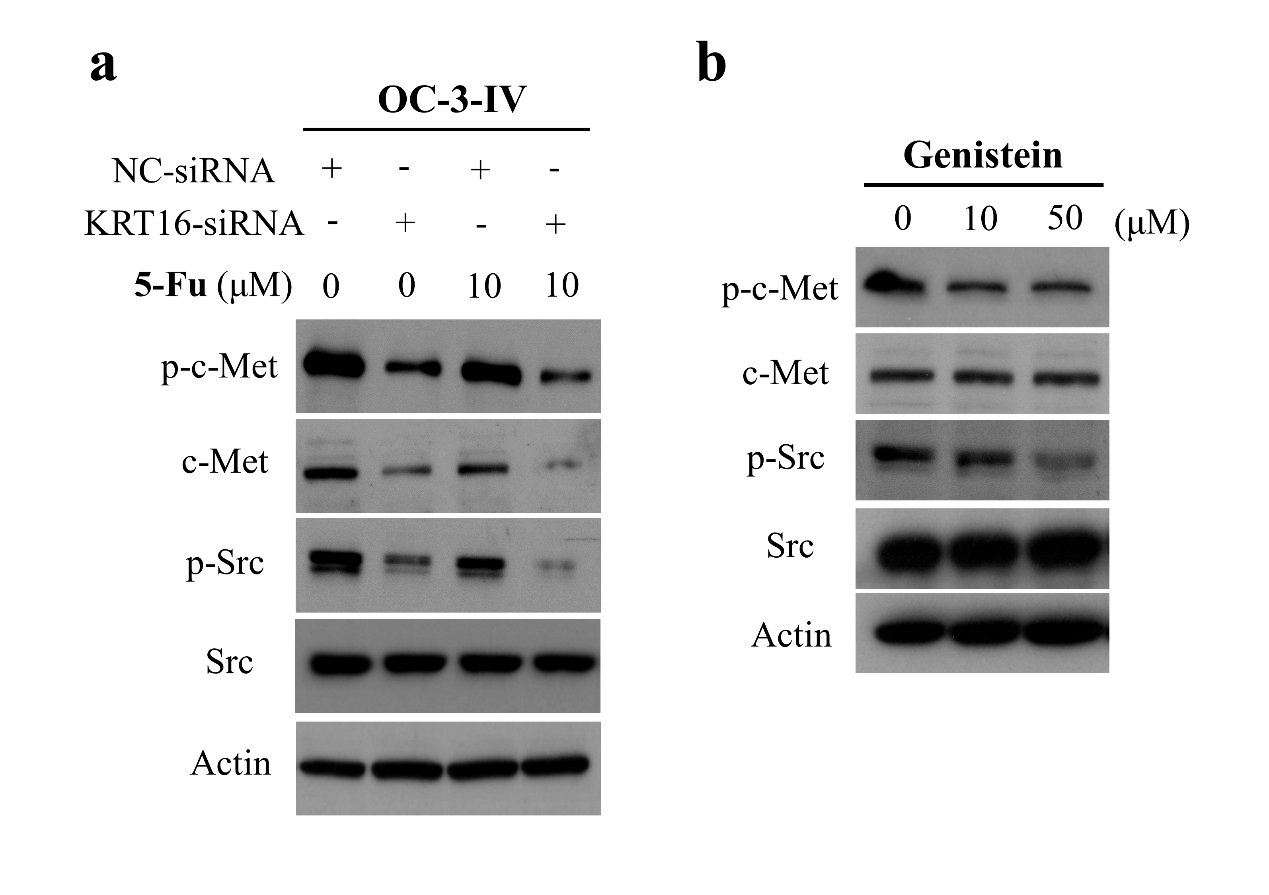


Figure **S18.** 5-FU and genistein inhibited activation of c-Met/Src signaling in OC-3-IV cells.

**(a)** The protein expression levels of the signaling components of the c-Met/Src pathway in OC-3-IV cells transfected with the indicated plasmids are shown by Western blotting. **(b)** The protein expression levels of the signaling components of the c-Met/Src pathway in OC-3-IV cells are shown by Western blotting.

**
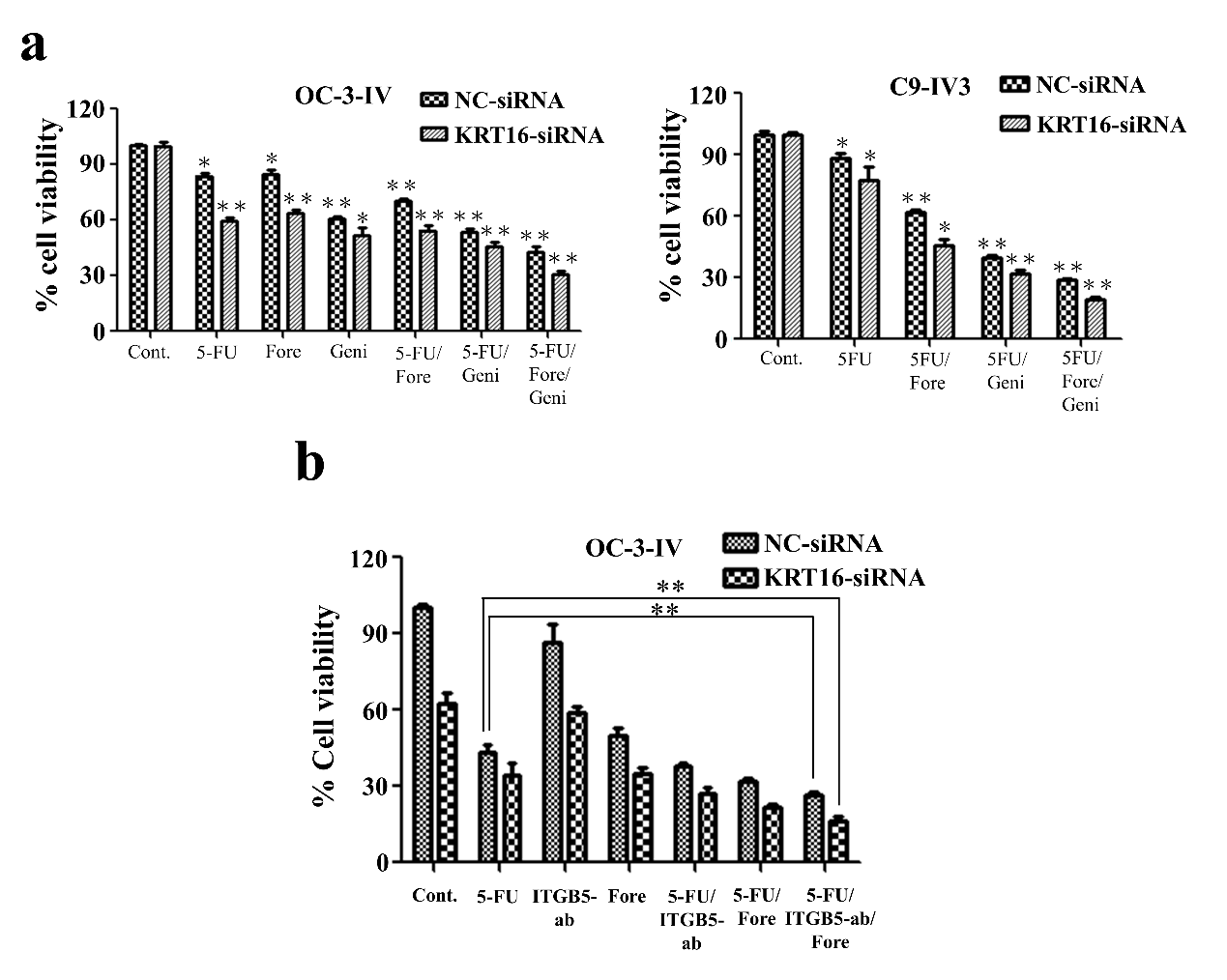
** Figure **S19.** Inhibition of KRT16/β5-integrin/c-Met signaling enhances cytotoxicity of 5-FU treatment in OSCC cells.

**(a)** Combination of 5-FU, foretinib (Fore) and genistein (Geni) or β5-integrin antibody (ITGB5-ab) increased cytotoxicity in OSCC cells. **(b)** KRT16 knockdown further reduced the IC_50_ of various combinations of drug treatments. Histograms represent means ± SD from three independent experiments (*, *P* < 0.05, **, *P* < 0.01).

**Additional file 1**

**Table S1.** Primers and siRNAs used in this study.

**
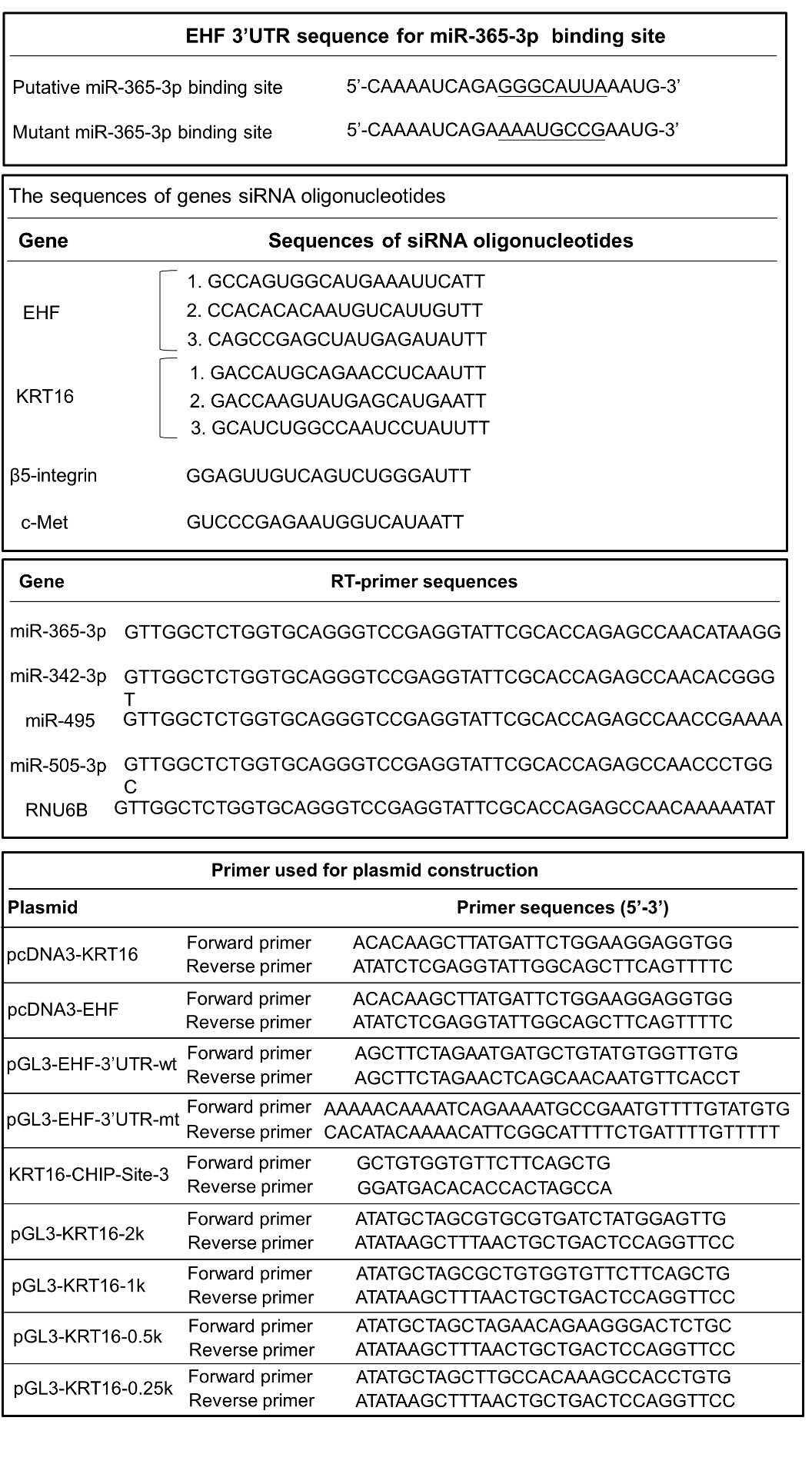
**

**Table S2.**Primers used in this study.

**
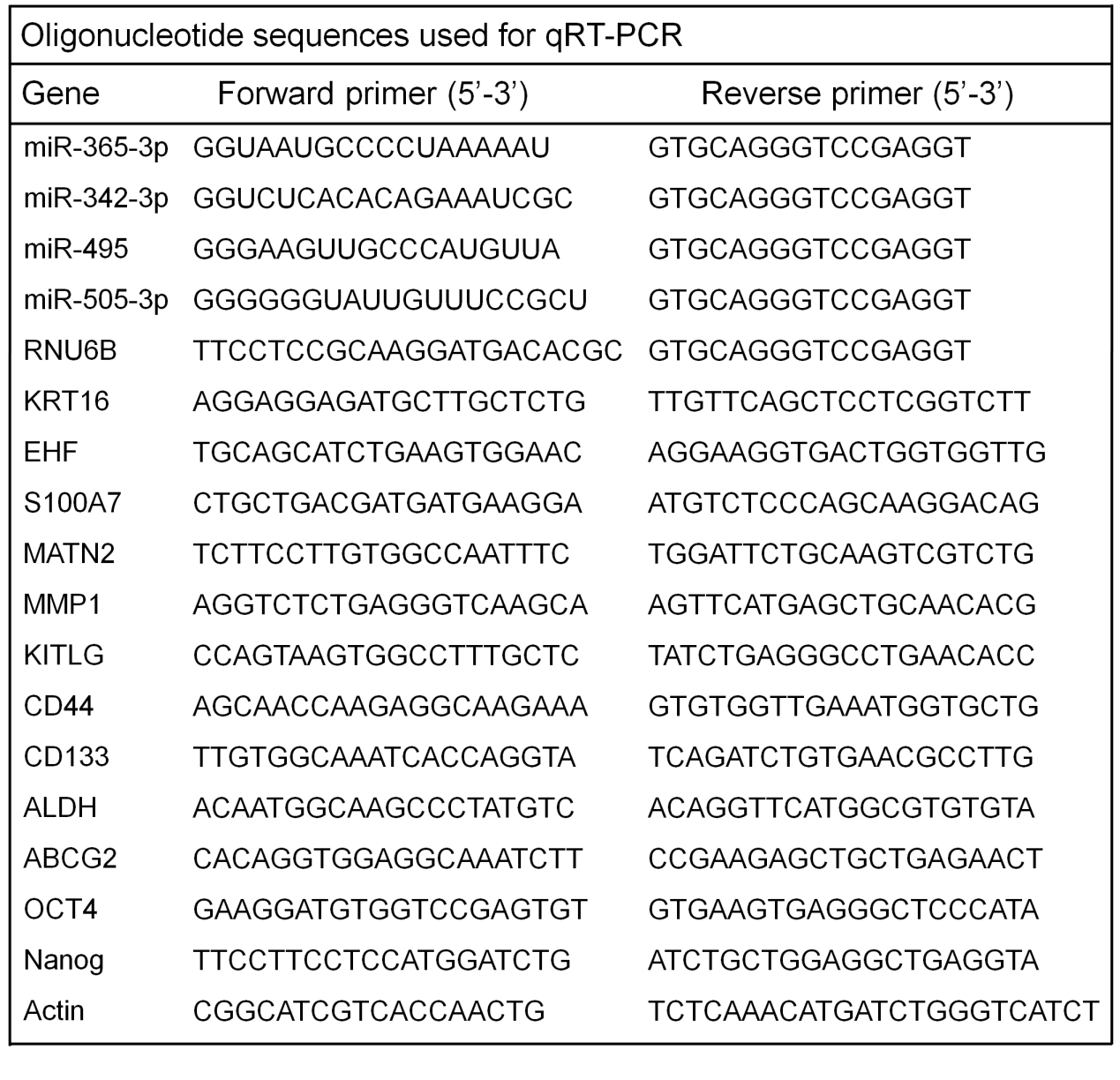
**
